# Engaging chefs in sustainable practice using Theory U to address greenhouse gas emissions and food waste

**DOI:** 10.3389/fsufs.2025.1641960

**Published:** 2026-01-05

**Authors:** Andrea Zick, Amber Lawes-Johnson, Ximena Schmidt Rivera, Christian Reynolds

**Affiliations:** 1 Department of Chemical Engineering, Brunel University London, Uxbridge, United Kingdom; 2 Department of Pathobiology and Population Sciences, Veterinary Epidemiology, Economics and Public Health, Royal Veterinary College, London, United Kingdom; 3 Centre for Food Policy, School of Health and Medical Sciences, City St George’s, University of London, London, United Kingdom

**Keywords:** participatory action learning and action research, menu engineering, food system actors, sustainable transformation processes, organisational change, hospitality and food service

## Abstract

**Introduction:**

This pilot study explored the use of Theory U as a framework for participatory workshops with chefs, aiming to support menu changes that reduce greenhouse gas emissions and food waste. The primary objective was to assess the feasibility of collecting and analysing data on chefs’ perceived barriers and opportunities for sustainable menu transformation. A secondary objective was to examine whether the workshop facilitated transformational learning loops among participants.

**Methods:**

A single participatory workshop was conducted with trainee chefs, applying Theory U principles to guide discussions and activities. Data were collected on chefs’ perceptions of sustainability, decision-making priorities, and influencing stakeholders. Qualitative analysis focused on identifying evidence of transformational learning.

**Results:**

The workshop generated rich qualitative data that can be linked to broader research on food systems and chef-led change. Findings revealed multiple factors influencing menu transformation, including stakeholder roles and current prioritisation practices. Evidence of transformational learning loops was observed; however, no clear indicators of transformational change emerged.

**Discussion:**

The study demonstrates that Theory U-based workshops are a practical entry point for engaging chefs in food system awareness and co-creating strategies for sustainable menu transformation. While promising, follow-up workshops are needed to validate these findings and refine data collection and analysis methods.

## 1 Introduction

Food systems researchers (Hasnain et al., 2020; Kok et al., 2019; Richardson and Fernqvist, 2022) agree on the need for transformation to address a poly-crisis of poor health (Thomas et al., 2024) and biodiversity (Dasgupta, 2021) outcomes combined with decreased food systems resilience (Lang et al., 2025). These food system outcomes are seen as a threat to future human and non-human generations, democracy, and peace (Dasgupta, 2021; Lang et al., 2025; Thomas et al., 2024). Understanding how food system drivers (e.g., socio-cultural, infrastructure, and technology) influence components such as the food environment is essential (Brouwer et al., 2020). This research adopts the systems-oriented view of food systems analysis proposed by Brouwer et al. (2020). It aims to respond to the call for a transition from concept to practical application for one component of the food system, the Hospitality and Food Service sector (HaFS). Such research requires the approaches described by Kok et al. (2019) as second-order research. By working within a learning and knowledge generation context, such as participatory action learning (PAL), one may also have the potential to achieve a double-system transformation, as it addresses the food systems’ and education systems’ need for changes to chefs’ training (Cappelen and Strandgaard Pedersen, 2024; Deutsch, 2016).

This study engaged chefs, key stakeholders in HaFS, through a workshop to co-create an understanding of menu transformation and explore menu design practices in this part of the food system. It was expected that this would lead to active and potentially transformational learning of chefs about the impact that decisions in menu creation can have. In addition, by focusing on HaFS, the call for analysing out-of-home consumption was addressed (Brouwer et al., 2020), and by investigating the decision-making and perspectives of chefs in particular, it was hoped to shed light on feedback loops within the menu and food offer transformation in HaFS and help to answer the ‘how to’ transform (Kok et al., 2019) menus.

Due to limited evidence of participatory workshops in these contexts, their suitability for research and co-creation was uncertain; thus, a review of similar interventions was necessary to test the approach by delivering the content to a group of chefs.

The objectives of this paper, therefore, are:

1) Describe the underlying epistemological assumptions of the workshop
2) Share the creation process, outline the content and review the feasibility of the workshop
3) Discuss the Theory U process and learning loops for transformational change in the context of the workshop evidence
4) Discuss the possibility of gathering research insights linked to menu and food offer transformation with the workshop artefacts.

Current food system transformation initiatives linked to HaFS suggest that menu and food offer changes can serve as an entry point for shifting diets to reduce greenhouse gas emissions (GHGE) and food waste (Pollicino et al., 2024). Pollicino et al. (2024) list six behaviour change categories relevant for diet transformation and the number of research trials in each of these categories: product (*n*-66), presentation (189), people (*n*-10), promotion (*n*-374), price (*n*-62) and placement (*n*-87). Notably, ‘people’ appears to have the lowest number of trials currently, and training chefs was seen as the most promising intervention within this category; this pilot design adds to this category. Most frequently, the outcomes of such interventions are assessed based on the barriers and opportunities perceived by consumers and HaFS representatives (Pollicino et al., 2024). However, suppose the change of menus is conceived through the lens of transformational learning and transformational change. In that case, there is an opportunity to explore the applicability of Theory U (Presencing Institute, 2024) as a framework for transformational learning and use Poutiatine’s (2009) nine principles of transformational change to assess such learning loops. The menu creation process, therefore, would be at the heart of these workshops, allowing chefs to explore this process and the proposed transformation.

In HaFS, menus not only list the food items a business offers but also fulfill several functions. They inform customers of available options, including their contents, prices, nutrition information, and allergen details (Mutlu et al., 2022). They are also a sales and marketing tool and, as such, support the business concept and brand image (Him and Chark, 2023). They play a key role in the commerciality of restaurants, as they influence profit margins. For example, carefully edited menus will often include loss leaders and highly profitable dishes (Mutlu et al., 2022). Furthermore, menus also reflect legislation and policies (Nie et al., 2024). In this research, they are viewed as an organising principle that can influence kitchen practices by defining which products, equipment, team members, style of cooking, and dishes are agreed upon for extended periods. It is interesting that the above attributes almost exclusively link to what the menu means for others, not involved in the daily production of the physical output.

While consumer-facing research focuses on choice editing and nudges (Pollicino et al., 2024) another school of thought also recommends editing the composition of dishes, specifically by decreasing animal proteins to align menus closer to the EAT-Lancet recommendations (Willett et al., 2019; WWF, 2023). Shifting the menu content, as the latter proposal for sustainable eating influences internal business practices, while nudges and choice editing suggest that consumers drive change. From a systems-oriented perspective, we argue that both can be true; however, the intervention with the most potential for transforming food systems is changing what is on the menu and creating offers with fewer dishes containing animal proteins (Forum of the Future, 2020; Guimarães et al., 2024; Seo et al., 2023). Such a menu change can reduce GHGE (Speck et al., 2022; Willett et al., 2019). When combined with reducing food waste, the decrease in GHGE can be further enhanced (WRAP, 2021a, 2021b). While there are other perspectives on sustainable menu transformations, such as the impact of diets on water scarcity (Hess et al., 2016), this research explicitly examines how changing menus towards lower GHGE and reduced food waste creates barriers and opportunities from a chef ‘s perspective.

The transition of the HaFS sector towards food offers lower in GHGE and food waste can be seen as an evolution from previous ways of menu engineering and writing, because considering the environmental impact of foods within a menu has not routinely been a component of menu engineering decisions. Interviews (Zick et al., 2025) revealed innovation opportunities as chefs struggled to make sense of GHGE linked to food. A participatory workshop on menu creation offered a chance to explore how chefs create meaning around sustainable food (SF) and make sense of GHGE linked to food production and consumption.

Despite growing interest in engaging chefs to reduce GHGE, academic evidence remains limited (Zick et al., 2024). Initiatives such as Climate Smart Chefs (Martin et al., 2023a; Martin et al., 2023b), the Chef ‘s Manifesto (Richardson and Fernqvist, 2022), Guardians of Grub (WRAP, 2018), Great Taste Zero Waste (EIT Food, 2024), or roundtables hosted by the Sustainable Restaurant Association (Huang and Hall, 2023). These campaigns highlight the growing importance of evaluating effective ways to engage with culinary professionals.

We propose that reducing food GHGE and waste requires transformational change (Poutiatine, 2009) and experiential learning, as the environmental impact of food is a relatively new and complex concept to which chefs have not previously been exposed. Poutiatine (2009) describes nine principles of transformational change. We adopt the framing of Poutiatine (2009),which views transformation as a cyclical process of learning, reflection, and applying a change in practice, leading to a new learning cycle. Most importantly, transformation requires second-order change, a departure from past practices, skills and knowledge evident in the earlier part of this research. Interviews showed that many participants struggled to explain GHGE in food and identify reduction strategies (Zick et al., 2025). While participants who have grasped the concept have started piloting and innovating (Perez-Cueto, 2021), it is not yet mainstream and thus, reduced GHGE and reduced food waste can be used as a unique selling point (Alvarenga Nascimento, 2023). This area, therefore, is a space for transformation and innovation. If the workshop enables transformational learning or change, it should be possible to find evidence for any or all of the nine principles described by Poutiatine (2009).

Participatory action research (PAR) methods have engaged citizens in food and science topics, offering co-creation and learning opportunities (Kluczkovski et al., 2020; García-Guerrero and Lewenstein, 2023). While these activities can be initiated through research outreach, they also present important opportunities for co-creation and experiential learning for both participants and researchers. Such projects provide access to places and people who might not otherwise explore the topic at hand or engage with scientific subjects (García-Guerrero and Lewenstein, 2023).

Theory U, developed by Scharmer and the broader U-Lab community, builds on PAR and has been applied in diverse fields, from education to FinTech, due to its flexibility (Presencing Institute, 2024). It differs from other transformational change theories, such as Lewin’s Change Management Model (Hussain et al., 2018), Ajzen’s Theory of Planned Behaviour (Yuriev et al., 2020) or Appreciative Inquiry by Cooperrider and Srivastava (Breslow et al., 2015; Bushe, 2013). Theory U acknowledges that individuals in a transformation process must explore and connect to their inner world to let go of what is and open their hearts and minds to what could be. It acknowledges that the awareness of the dissonance between the expected and experienced outcomes evokes fear for most people, and this fear needs to be overcome to trial and innovate. The desire to innovate and be creative among chefs, frequently mentioned by participants in (Zick et al., 2025), suggests that groups of chefs would receive this approach well, as it taps into their curiosity and playfulness.

Theory U guides individuals from system awareness to personal transformation, encouraging collaborative innovation through shared vulnerability (Scharmer, 2016). Numerous studies prove that humans find change difficult, and various reasons exist for why practice change is challenging (Chawla and Lugosi, 2025; Hebinck et al., 2018; Hussain et al., 2018). Theory U proposes that for a person to be willing to change, they need to connect to their internal source, allowing them to imagine a different future (Scharmer, 2016). The process of letting go of known ways of doing things can be more straightforward when done in collaboration with others in the system, as it highlights that others are facing the same insecurity and allows for the pooling of resources (Scharmer, 2016). Most complex system changes are challenging and require a shift in system paradigms and worldviews, but more frequently, processes and structures are redesigned rather than departing from underlying paradigms (Scharmer, 2022). This paradigm shift happens at the bottom of the U process, creating new mindsets and allowing space for prototyping and innovation.

Critics including Heller (2019) and Vardaman (2020) argue that Theory U oversimplifies change and lacks specificity. Still, both acknowledge its value in leadership and education. According to Heller (2019), the oversimplification of societal change is presented as a linear process, which indicates socio-political naivete and leads to incoherent principles. However, Heller (2019) endorsed Theory U as a non-conventional school of thought regarding leadership and innovation, emphasising emotional intelligence and transdisciplinary. Heller (2019) believes this can be useful in education and organisational learning and poses strong potential for transformation and change management.

Vardaman (2020) critiques Theory U as vague and overly idealistic, echoing Heller’s (2019) concerns. Both see their value in leadership development, particularly in shifting from ego- to eco-centric perspectives. This study tests Theory U in menu engineering, aiming to build chefs’ systems awareness around GHGE and food waste. Bronfenbrenner’s Ecological Systems Theory (Neal and Neal, 2013) can be used as a lens to explore how chefs, as one actor in this interconnected and nested food system (Zick et al., 2024), shape it and how their practices are being shaped in it. The conceptual framework proposed by Bronfenbrenner (1995) and Bronfenbrenner and Morris (2007) suggests that people can be mapped into macro, exo, meso, and microsystems. Within the workshop, we utilised these dimensions and integrated them into the Theory U process to enable chefs to become aware of these dimensions, thereby facilitating systems awareness (Scharmer, 2022) and navigating access points for change.

This is a novel application of Theory U and PAR in HaFS, contributing to organisational change research and offering insights for hospitality and gastronomy scholars. The study also highlights micro-level interactions in menu transformation and identifies practical barriers and opportunities and thus may inform food systems researchers of wider food systems interactions.

Stakeholders struggled to understand the embedded GHGE (Zick et al., 2025) linking it to concepts such as the impact of transport or energy used in the business. However, this incomplete knowledge could serve as an anchor point for transformational learning. Dialogue with peers and collaborative sense-making could enhance a reassessment of this incomplete knowledge about embedded GHGE. According to Navarro (2006) people adopt new knowledge through experience, dialogue, and reflection, key elements of PALAR (Wood and Zuber-Skerrit, 2013).

We theorise that menu transformation requires experiential learning and transformational change because the environmental impact of food is a new and complex concept to which chefs have not previously been exposed. More importantly, to enable kitchens and catering businesses to incorporate this new dimension into their practices, emerging futures must be envisioned and considered as a pathway to start testing different approaches, allowing the Theory U approach to be applied in this context. A pilot workshop was designed to test Theory U’s feasibility in embedding GHGE and food waste knowledge into menu creation and to assess evidence of learning and systems awareness. The aim was to fill a practice gap in the catering sector by incorporating knowledge about GHGE embedded in food and food waste into the menu creation process and to assess whether there is evidence of transformational, experiential learning, and systems awareness after the workshop.

This paper outlines the workshop design, methods, and analysis of artefacts, contributing to food systems transformation research and exploring how sector–academic collaborations can foster transformational learning.

## 2 Materials and methods

The following sections describe the ethical and methodological considerations, as well as the study limitations (Section 2.1), the development of the workshop (Section 2.3), and its framing within Theory U (Section 2.4). Lastly, the workshop outline and set-up will be shared (section 2.4). Appendix 3 outlines the workshop’s underlying theoretical approaches, including PAR, PAL, and Theory U, to supplement the methodology.

### 2.1 Ethical and methodological considerations and limitations

In the lead-up to the pilot workshop, the researcher explored various ethical considerations, detailed in Appendix 2. Ethics approval (44491-LR-Aug/2023–46,866-1) was received at the end of August 2023.

The decision to deliver a workshop as a research approach was influenced by the previous practical experience of the lead researcher (LR), who had worked in the HaFS sector as a chef and subsequently led various sustainability projects alongside chefs. A scoping literature review conducted in October 2022 on research involving chefs revealed that few studies had utilised focus groups or workshop-like events when working with chefs to explore research questions (see Appendix 1).

The scoping literature review (Appendix 1) indicates that interviews were the most used method in previous research on topics related to chefs, followed by literature reviews of media and academic papers and extensive surveys. Mixed methods, which often combined interviews with other methods, such as surveys or ethnography, were also prevalent. The most similar method to workshop delivery in the data was focus groups; however, only two studies employed this method, and two further mixed-methods studies included focus groups as well. At a later stage of the research process, a study was reviewed that utilised PAR in German care catering settings to reduce food waste (Strotmann et al., 2017a; Strotmann et al., 2017b). This project suggests that PAR could be a valuable method for understanding the context of systems change.

Finally, the early scoping study of (Zick et al., 2025) found that chefs prefer in-person interactions over online surveys. There is anecdotal evidence (Sommerlad, 2023) that individuals with ADHD are more prevalent in the hospitality industry, particularly in kitchens. For example, some ADHD symptoms have been reported to be conducive to working in hospitality and kitchens, such as less tedious application processes, the requirement to be creative and active and workaholism (Adamou et al., 2013; Hotte-Meunier et al., 2024). This adds to this anecdotal evidence, but prevalence statistics are yet to be established. Research methods requiring chefs to complete lengthy surveys or those perceived as static may limit research engagement. The frequently mentioned time famine of chefs (Zick et al., 2025) suggests that chefs are more likely to engage with research if they perceive value in such involvement. Therefore, supporting them in learning about a subject that is increasingly becoming relevant in the sector (GHGE of food and food waste reduction) and is seen as a unique selling point was believed to be an enabler for participation. This was further enhanced by the need for businesses to understand this subject.

PAR methods differ significantly in the extent to which the researcher influences the study. These methods acknowledge that the researcher influences the context being studied. Many PAR researchers may argue that the intent is to co-create solutions to complex challenges; thus, the researcher is an integral part of the process (Birney et al., 2025; Scharmer, 2016). The embeddedness of the researchers within this process is also one of its most common critiques, as the biases of the researcher will be carried into the process, leading to power imbalances, also framed as ‘elite co-option’ (Keahey, 2021). This means the researcher’s position needs to be considered in terms of the ethics application, the project design and delivery, and the data analysis. Therefore, it requires detailed records of the project development, delivery, analysis, and outputs, as well as a process of reflexivity.

Camilleri et al. (2023) demonstrated that most people stop engaging in active communication when in groups of more than six people. This can be navigated by dividing a larger group into subgroups of up to six people, with at least one facilitator assigned to each group. Thus, the ratio of participants to workshop facilitators would be 1:6 or less.

### 2.2 Pilot workshop development

Between July and mid-September 2023, the LR and a research assistant (RA) co-created and then facilitated the workshop. The RA was engaged to invite a greater number of participants (12–16) and to allow a second ‘research opinion’ in the room.

A host business was utilised for the pilot to facilitate the recruitment process and to gain insight into the business’s perspective on such an initiative. Recruitment and engagement with the host business began approximately 6 months before the workshop. The LR delivered three engagement sessions, each lasting around 40–60 min, and involved the restaurant management, human resources business partner, and chefs (~ 10 from junior sous chef to executive chef level). The content of the pilot workshop was developed over 2 months, which included a site visit and approximately four online sessions between the researchers to review the content and delivery of the workshop’s various sections.

The LR proposed the structure and content of the workshop, which were then developed iteratively among the workshop development team, comprising researchers, project supervisors, the executive chef, and other business representatives. This iterative approach was employed to mitigate the LRs positionality bias, given the LR’s employment within the hospitality industry. The development team questioned and suggested methods and terminologies during the development process. The LR reflected on the purpose of each chosen subject, the design and the timelines. Records of adjustments made in response to ongoing feedback and critique were maintained for the follow-up workshops and the subsequent interim report of the project.

Follow-up interviews with workshop participants and the RA were recorded and transcribed with MS Teams. The LR conducted a reflective interview, responding to the same questions as the participants and the RA. This was also recorded and transcribed in MS Teams. This additional data was thematically coded for DT and sentiment shared in Table 1.

**Figure 1:**
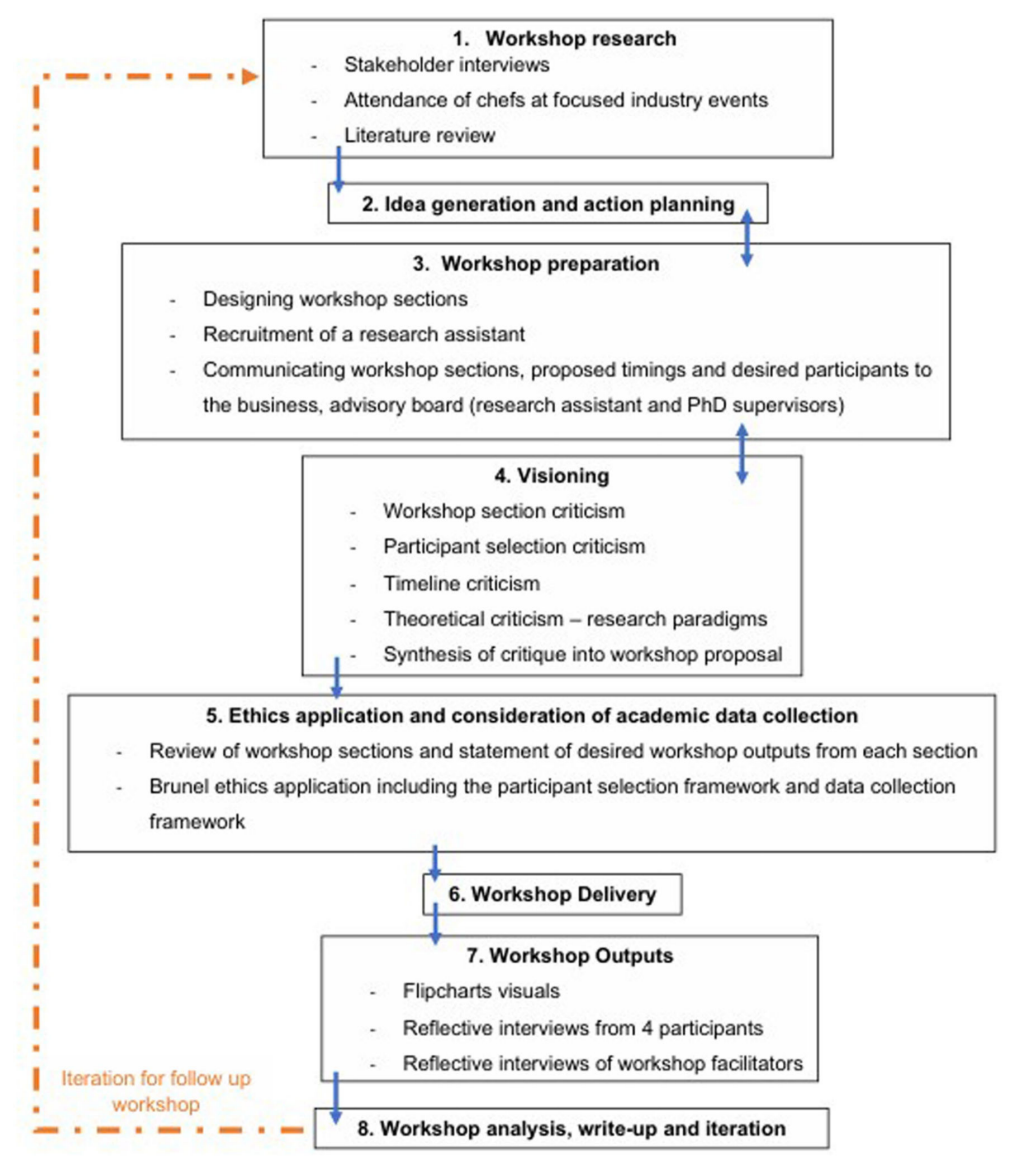
Workshop Design Process Numbers and blocks refer to each step of the process, while arrows describe the sequence between the steps, including iteration loops between steps. The dotted arrow symbolises the iterative aspect of the process between workshops.

Within the workshop’s development, reference was made to the stakeholder interview themes (Zick et al., 2025). The overarching workshop process was guided by Theory U (Presencing Institute, 2024). The content of the workshop sections also draws on photo voice methodology (PhotoVoice, 2025), research informed by IFSTAL (Pope et al., 2021), and systems change facilitation practices acquired through the Spark course at the School of Systems Change (School of Systems Change, 2025). Figure 1 summarises the workshop development process.

The design was grounded on guiding the participants through the process of Theory U: seeing, sensing, presencing, and crystallising. Theory U offers a range of tools, but these were perceived as not being tailored explicitly to catering contexts. The LR visited other events targeted at chefs and culinary colleges to observe how chefs engaged with these events. Where possible, reflective summaries were written, and official records of the events were kept. This further supported the thinking behind the workshop design and how to guide participants through the Theory U process.

### 2.3 Theory U framing–selection of workshop themes and structure

These were guided by the findings of stakeholder interviews (Zick et al., 2025), consultation with the business that supported the workshop’s delivery, participation in and reflection on targeted events for chefs, and the research question.

The Theory U toolkit (Presencing Institute, 2024) was adapted for the specific context. The structure was built with participants in mind, allowing them to go through the U process within the workshop context and leaning towards transformational, experiential learning theory and dynamic skills theory. Furthermore, as Theory U aims to generate systems awareness, a previous study was employed to consider different systems dimensions for chefs (Zick et al., 2024) across the various workshop sections.

Table 2 lists each section of the workshop, its purpose, and relevance to Theory U, as well as the system’s dimensions, based on Bronfenbrenner’s ecological systems theory, that were addressed.

### 2.4 Workshop outline and set-up

The complete outline and planned timings for the workshop can be found in Appendix 4, Table 8. The total active time of the workshop was anticipated to be 4.5 h, with sufficient breaks to give participants time to reflect and embed what they had learnt.

Participant information sheets, consent forms, and pre-workshop work were sent approximately a week before the workshop. Everyone was asked to email one photo showing ‘good food’ (GF) and one picture they associate with ‘sustainable food’ (SF). Randomly printed food-related images (46) from Pexels (2025) were available on the workshop day as a contingency for participants who did not pre-submit images. A list of equipment and materials for the workshop is provided in Appendix 5.

As we aim to conduct further workshops, we also considered how the workshop artefacts (the flipcharts and images created during the workshop) could be systematically analysed; this can be found in Appendix 6. A comprehensive discussion of the artefact analysis is not intended within this paper; however, considering how the collected data could be analysed was part of the workshop development. Therefore, we review the feasibility of such an analysis in Section 3.2, where we share examples.

## 3 Results

The results present first descriptive characteristics of the participants in Section 3.1, the workshop outputs or artefacts in Section 3.2, including a feasibility and practicality evaluation in Section 3.2.1, and a brief descriptive summary of the artefact and reflective interview analysis in Section 3.2.2. The results section finishes with an assessment of the workshop timings in section 3.2.3.

### 3.1 Descriptive results

The researcher invited 26 chefs (six females and 20 males), and nine agreed to participate. However, only eight chefs participated in the workshop on the day. Further, one of the participants arrived after the welcome section and the ‘GF’ and ‘SF’ reflections. A summary of participants’ demographics and sector-related information is displayed in Table 3.

### 3.2 Workshop outputs

The 25 workshop artefacts created during the event are listed in Appendix 6, along with the proposed analysis protocol. A detailed thematic analysis of the artefacts will be carried out in the future and reported as a separate study. Here, the feasibility of the analysis is prioritised rather than the meaning of the uncovered themes. Workshop Artefact 1 displays the agreed-upon ways of working with the group during the workshop. This activity was completed as a group, following the photo-elicitation for ‘good’ and ‘sustainable’ food.

Five participants completed the pre-workshop task, submitting two images: one representing ‘GF’ and one representing ‘SF’. This meant the facilitators provided a selection of printed images linked to food from Pexels (2025) used to choose from on the workshop day to enable everyone’s participation. One of the three who had not completed this task was the participant who arrived late, and thus, only two participants used this method to choose a suitable reference image.

Appendix 6, Workshop Artefact 2–7 display the photos of the flipcharts, which were used to display pictures and capture the captions and narratives shared by each participant. Names were removed (white areas).

The chefs’ ecosystem, menu hierarchy, and planet-kind dish sections of the event Appendix 6, Workshop Artefact 8–10 were facilitated in two groups of four people. The participants remained in their group for all three parts.

The Blue-Sky thinking activity is captured in Appendix 6, Workshop Artefact 15–19. Further data were collected during the workshop debriefs of Workshop Artefact 20 and 21. Lastly, any changes made in the workshop due to time constraints were documented in Workshop Artefacts 22–25.

#### 3.2.1 Workshop feasibility and practicality evaluation

To assess the feasibility, we report on participant recruitment and attendance, the time required to prepare and deliver the workshop, which sections yielded expected and unexpected outcomes, and how we attempted to analyse the workshop artefacts. Following this, we share the feasibility and observations from the workshop section by section, along with summaries of the artefact analysis for each workshop section.

The in- and post-workshop debriefs were analysed by sentiment, perceived ease, and recommended changes. These are displayed in Appendix 15, Table 15. The LR and RA generated these ratings from the review of the in- and post-workshop debriefs. This analysis was used to assess the feasibility and practicality of each workshop section, as shown in Table 4. The rating was overwhelmingly positive, with no ratings lower than 2 (out of 5), confirming the overall feasibility and practicality.

##### 3.2.1.1 Welcome, and creation of a ‘safe’ environment and ground rules

Participants found setting the ground rules easy and felt it was a beneficial start to working together for the day. However, it took longer than anticipated, so giving fewer prompts may counteract the perception that it is a burden, leading to disengagement. Appendix 6, Workshop Artefact 1 displays the agreed ground rules for the day.

##### 3.2.1.2 ‘Good’ and ‘sustainable’ food photo elicitation

All participants engaged actively and used the opportunity to share their positionality through food; two participants selected their images on the workshop day and were able to participate in the conversation. Appendix 6, Workshop Artefact 2–7 display the images and the captions discussed.

##### 3.2.1.3 Researcher presentation about food waste and GHGE of food

The PowerPoint presentation, which was used to share information on GHGE and food waste relevant to chefs, was the only part of the workshop not designed to be co-created and used purposely for data collection. Both facilitators ensured they checked back with the group and allowed for questions to be asked throughout, leading to various linked conversations about cattle farming practices in the UK and current food waste practices in the chefs’ workplace. The task of calculating the associated emissions appeared to be eye-opening. GHGE contribution for a simple shortbread recipe (selected by the participants) was assessed using a free online tool from MyEmissions (2025). Once the first calculation was made, the recipe was reformulated, and butter was replaced with margarine. The GHGE were recalculated, leading to a significant decrease in the recipe’s embedded GHGE. This led to a lively discussion among the group about this swap, with one chef questioning the health implications of the change, for example.

##### 3.2.4.4 Creating menus–chefs’ ecosystem–fuzzy cognitive mapping

The task required some time for familiarisation; however, the printed stakeholder prompts allowed them to start exploring who influenced the food offer quickly. Having a facilitator in each group meant the conversation could be captured without the participants feeling concerned about catching the meaning and findings of their group exploration. Additionally, facilitators ensured that all voices were heard by asking follow-up questions when necessary. Appendix 6, Workshop Artefact 8–9 show this task’s co-created stakeholder maps and facilitator notes.

##### 3.2.4.5 Menu onion/ladder–menu priorities–group deliberation

Some discussion on the priorities placed on menu decisions began in the previous workshop section. However, with this standalone section to follow, the participants had more time to explore the priorities of menu decisions. The prompts created from the academic evidence helped focus the group on the task and question. There were lively discussions on which menu aspect was most relevant, with some suggested prompts seen as equally important. The number of prompts, however, made it hard to complete the task on time, so overall, the activity felt more rushed than the other parts. Appendix 6, Workshop Artefact 11 and 12 represent the group’s deliberations on this task.

##### 3.2.4.6 Creating a sustainable dish

This section appeared to challenge the participants, possibly due to the openness of the task. Overall, the engagement and conversation progressed less fluidly. The facilitators were required to ask questions so the participants would explore the topic with more interest. Appendix 6, Workshop artefact 13 and 14 record both groups’ work from the event.

##### 3.2.4.7 Blue-sky thinking–backcasting

Appendix 6, Workshop Artefact 15–19 depict the results of this workshop activity. This section was partially individual; all participants were asked to write suggestions. Everyone contributed to the flipcharts with actionable ideas to drive forward food waste and GHGE reduction within three timeframes (3 months, 1 year, and 2030). All participants then voted for their favourite suggestions from the group, and this was followed up with a generative dialogue about the most popular choices, helping the chefs reflect on the solutions they felt they had control over.

##### 3.2.4.8 Reflections and feedback

This session felt rushed due to the time constraints of starting later than anticipated and overrunning in other parts of the workshop. Further, the participants were visibly tired and less focused. However, allowing direct and instant feedback was adopted by some participants and remains potentially valuable. Appendix 6, Workshop Artefact 20 and 21 contain the facilitator’s notes from this generative panel session.

##### 3.2.4.9 Graduation–gifting of Carlin pea sprouts and a certificate

The unexpected ‘awards ceremony’ included receiving a certificate of completion and a Carlin pea plant to symbolise that the workshop was the beginning of planting a seed of ideas and solutions that will need tending to by everyone in the group. This offering was received joyfully by all participants. While the certificate was not accredited with continuous professional development programmes, it reinforced the message that everyone contributed to the knowledge created and was a means of thanking the participants for their insights.

##### 3.2.4.10 Follow-up 1 to 1 reflective interviews or questionnaires

Three out of eight attendees took the time to complete the reflective interviews or questionnaires, which was below the desired number of six. However, the LR had opportunities to speak to most participants after the event on other occasions, so where there was a lack of engagement in the formal feedback, informal feedback was received from most participants.

#### 3.2.2 Workshop artefact analysis

This section summarises the protocol for analysing various artefacts. The purpose of sharing this data here is to illustrate how it can be systematically examined. The findings of this preliminary analysis will be reviewed and discussed in a future paper.

##### 3.2.2.1 ‘Good’ and ‘sustainable’ food photo elicitation

We proposed creating Appendix Table 9 for ‘GF’ and ‘SF’ to explore recurring themes and narratives inductively and to compare our findings with those of studies such as Diaconeasa et al. (2022) and Mapes and Ross (2022). The participant descriptions from the chosen images Appendix 6, Workshop Artefact 2–7 are listed, and GF and SF are compared for each participant. We captured whether the picture was selected from their images or Pexels.com. Organising the image descriptions in this table makes it possible to systematically compare narratives of good and SF within the group and participant differences.

##### 3.2.2.2 Creating menus – chefs’ ecosystem - fuzzy cognitive mapping

For this task, we suggest creating a table that lists actors chosen by each group in descending order of relevance, along with a review of themes recorded from the generative dialogue to explore Inductive themes (IT). This task is rooted in concept maps and fuzzy cognitive mapping, helping to examine a group’s constructs of knowledge and worldviews.

Appendix 6, Table 10 summarises the choices of relevant stakeholders for both groups’ menu creation and indicates how influential or closely involved each group perceived these to be.

The prompts for the stakeholders were easy for the participants to understand. One of the groups created a rudimentary causal loop diagram Appendix 6, Workshop Artefact 9, indicating how different stakeholders influence each other and the menu ideation process. The generative dialogue of the panel conversation helped consolidate the importance of various stakeholders, as shown in Appendix 6, Table 11. Appendix 6, Workshop Artefact 8–10 contains all evidence collected from this task.

##### 3.2.2.3 Menu onion/ladder–menu priorities–group deliberation

The researcher drew on two academic articles (Mutlu et al., 2022; Ottenbacher and Harrington, 2007) to develop workshop activity prompts based on the menu decision priorities outlined in those studies. The groups could use the prepared prompts to review the priorities of menu decisions and create priority lists of what influences menu ideation from their perspective. Thus, the intention was to create a table organising the selected priorities of the prompts in the food offer creation process to compare and contrast them with those in the articles.

Appendix 6, Table 12 summarises the agreed-upon priorities for each group, and Workshop Artefact 11 and 12 show the collected data. The priorities are listed in descending order for each group in Appendix 6, Table 12. We have highlighted two priorities that are directly linked to food waste and its environmental impact. Notably, Group A placed less priority on these than Group B.

##### 3.2.2.4 Sustainable dish creation

For this task, we anticipated comparing the framing of suggested dishes and the approach each group took, highlighting any recurring, divergent themes and concepts. As well as potentially missing themes linked to SF by the participants earlier in the workshop and those found in this rapid review by Reynolds et al. (2022).

Both groups devised a planet-kind dish but used different approaches, as seen in Appendix 6, Workshop Artefact 13 and 14. Appendix 6, Table 13 summarises their ideation, including the dish name, ingredients, the chosen sustainability narrative, and facilitator comments from this activity.

##### 3.2.2.5 Blue-sky thinking–backcasting

This part of the workshop is based on research into participatory backcasting for sustainable futures (Remans et al., 2024). The Blue-Sky Thinking Workshop Artefact (15–19) can be compared with this article. The task’s voting mechanism helps to understand the priorities selected by participants for future action, with the narratives and themes explored earlier in the workshop.

Appendix 6, Table 14 summarises the data found in Appendix 6, Workshop Artefact 15–19. The participants developed several ideas on how they could directly contribute to the business’s sustainability ambitions. There were equal numbers of suggestions and ideas for a three-month and a year; however, there were fewer ideas for 2030. The darker the shade of grey in Appendix 6, Table 14, the more votes the idea gathered; therefore, it would be a preferred initiative by the participants.

##### 3.2.2.6 Reflections, feedback and ‘follow-up 1 to 1 reflective interviews’ or questionnaires

To analyse this part of the workshop, we proposed that the data collected from the group debrief at the end of the workshop, the workshop follow-up participant questionnaire and the reflective interview with the RA were thematically coded and used as evidence for the overall feasibility, practicality, and effect on transformational learning.

Due to increasing time constraints and participants becoming visibly tired towards the end of the workshop, this part was shorter than planned. Workshop Artefact 20 and 21 summarise the workshop feedback taken on the day. Appendix 7 lists in-person workshop debrief feedback and displays a summary table, including quotes from participants, that was requested 2 weeks after the workshop. Overall, their responses corroborated the value of the workshop; in particular, the pace and structure of the workshop were seen as suitable. Participants also confirmed that the workshop empowered them to see how they can support the business change the food offers and that being outside the kitchen allowed them to think about their work differently. Consequently, they were able to consider where change was possible. This was further elaborated on in the two-week post-event feedback. Three participants and the RA gave structured feedback after the event. They are summarised in Table 1. Overall, participants and the RA endorsed the workshop, and one of the participants elaborated on the sustainable dish activity: ‘*One of the dishes ideated was trialled the next day in the kitchen’*.

#### 3.2.3 Timeline assessment

Workshop Artefact 22–25 in Appendix 6 show the required adjustments to the workshop schedule due to time constraints. While both facilitators tried to maintain the planned pace, the workshop started later, and some sections took longer than anticipated. This meant fewer and shorter breaks were given with the approval of the chefs in the room, and some sections were shortened. For example, the first group activity was planned to start at 11:45 a.m., but did not begin until 12:45 p.m. The facilitators brought the workshop timeline back on track for the end of the day, so the debrief started at 4:15 p.m. as planned.

## 4 Discussion

This section of the paper discusses the feasibility of the workshop (Section 4.1), examines the Theory U process and learning loops for transformational change (Section 4.2), and concludes with a brief outline of the possible artefact analysis (Section 4.3) and the study’s limitations (Section 4.4).

### 4.1 Method feasibility–workshop

The time, logistics, and financial costs involved make this a high-risk research method. For instance, labour costs for eight chefs were estimated at £886.40 (based on the London Living Wage of £13.75/h). Thus, one must ask whether the quality of the insights is sufficient to answer a research question and identify a gap. Despite the costs, the process offered value; participants gained learning opportunities and action plans, while researchers benefited from engagement with industry and proof-of-concept insights. These aspects made this high-risk approach reasonable for all parties. Additionally, such engagement with a business allows researchers the potential to establish a network of advisors for future projects.

While all parties benefited, the method relies heavily on trust. Researchers had to manage expectations transparently, requiring additional time and resources. The workshop followed a learning-action cycle, consistent with PAR literature (Wood and Zuber-Skerrit, 2013) and required strong management support, which aligns with the findings of Strotmann et al. (2017b).

The researchers did not achieve the desired number of participants, 12–16, despite long recruitment and engagement activities and planning a suitable date with the business to maximise the number of participants. This limits the assumptions which can be drawn from the workshop’s findings. However, we compared it with the event attendance of more established organisations working in this field, such as Climate Smart Chefs (*n*-61 online) registered in focus groups in (Martin et al., 2023b) or roundtables of the Sustainable Restaurant Association [40 businesses in 4 in-person roundtables, Sustainable Restaurant Association (2024)]. Further, the LR attended three climate-smart chef online roundtables, and a summary of the events was shared afterwards. According to these event summaries, the attendance was as follows: *n*-46 (01.12.22), *n*-36 (08.02.23) and *n*-26 (26.04.23). Therefore, the attendance at this pilot workshop typically reflects the sector, especially considering that the organisation’s events referenced here had chefs and other sector representatives in attendance. These events further drew their participants from multiple businesses, and the Climate Smart chef roundtables were shorter and hosted online.

The small group size (*n* = 4) proved effective, generating rich discussions and content-dense artefacts, supporting Camilleri et al.’s (2023) recommendation of 4–6 participants.

#### 4.1.1 Participant workshop assessment

According to the evidence from the workshop feedback and reflective interviews, the overall perception of the event’s space, length, context, environment and relationality was rated positive, suggesting that a safe space for sharing personal views was created. Participants notably endorsed the chance to get to know each other through the ‘good’ food and ‘sustainable’ food activities, the idea-sharing opportunities, and the fidget spinners. These aspects created a sense of community and belonging.

### 4.2 Theory U process and learning loops for transformational change

#### 4.2.1 Guiding participants through the theory U process

The workshop aimed to guide participants through the Theory U’s awareness-based systems change (Koenig et al., 2024) by having them share their views on their experience of the microsystem and raise awareness about the contrasting expectations of the broader societal discourse of the macrosystem. In this case, the goal was to guide the chefs in the workshop to become aware of their context in the kitchen and restaurant, as well as the aspects that influence their decisions regarding menu and food offer creation. This allowed space to explore the current context, specifically the dissonance between some of the frames of GF and SF.

The ice breaker (Appendix 6, Workshop Artefact 2–7) fostered shared language and initiated system sensing by prompting participants to reflect on and compare ‘good’ and ‘sustainable’ food concepts with overlapping but distinct meanings. However, the boundaries between the two are fuzzy and can overlap. This exercise was reflective at the core because it asked participants to consider their views and share them with others. This created a space where commonalities and differences of ‘good’ and ‘sustainable’ frames became visible. Commonalities included specific food attributes, such as with whom the food is shared or/where it was produced. Participants explored how societal and institutional definitions of sustainability (exo/macro-systems) sometimes conflicted with personal views, illustrated by a debate on sustainable seafood and British fishing. Specifically, a perceived demand for chefs to support British fishing while preventing overfishing has led to an interrogation of what sustainable seafood might mean. From this deliberation process, it became apparent to the group that SF can be associated with social practices that may not be desirable but support environmental sustainability. The presentation on GHGE reduction (e.g., swapping butter for margarine) sparked further discussion on trade-offs between environmental, health, and local economic values.

Stakeholder mapping exercises (Appendix 6, Workshop Artefact 8–10) and priority tasks (Appendix 6, Workshop Artefact 11–12) shifted focus to mesosystem dynamics, helping chefs recognise the influence of other actors and the low prioritisation of sustainability in menu decisions. Their consideration of other actors and their role as chefs in the menu creation process led to an understanding of the need to engage with these stakeholders to create menus and food offers, as well as acknowledging that other stakeholders may have different demands on the menu. Furthermore, they realised that sustainability was relatively low on the priority list despite being something many found desirable personally. This dissonance between personal values and external demands served as a presencing moment (connecting to one’s source intuition) (Scharmer, 2016), prompting reflection on whether to innovate or maintain the status quo. The low priority of sustainability contradicts what they would want according to their values or what they perceive as the social norm in the current reality. At this stage, participants were faced with the choice of redesigning, reframing, innovating, or retaining the status quo (Presencing Institute, 2024) of the current menu and food offer priorities.

The aim of the workshop’s Planet Kind dish and Blue-Sky Thinking sections (Appendix 6, Tables 13,14) was to move participants into the crystallising, reframing and innovation space. This allowed participants to explore collaboratively what possible future pathways might emerge through co-creation (Presencing Institute, 2024). The Planet Kind dish activity aimed to spark innovation but proved challenging, possibly due to timing. One group quickly assembled a low-GHGE dish using provided materials, while another engaged in deeper discussion and proposed a pilot dish. The quickly assembled dish arguably did not consider other business needs, such as whether it would appeal to customers and align with the overall restaurant business concept; however, it was finished before the allocated time, which was unexpected. The other proposed a dish that was seen as a good ‘pilot dish’ (Appendix 6, Workshop Artefact 13–14), and the conversation indicated that they viewed it as a starting point for development opportunities and iterations. Their approach was more considerate of the needs of other stakeholders in this process. This group had more senior chefs as participants, so it is possible that their experience of menu development influenced these different approaches. The researchers perceived this to be the part of the workshop which delivered the least on its objectives.

The Blue-Sky Thinking task (Appendix 6, Workshop Artefact 15–19) generated numerous short- and mid-term ideas, but fewer for 2030. While voting helped prioritise proposals, deeper reflection was limited, possibly due to time constraints. The researchers did not observe deliberation and reflection among participants beyond those initiated during the panel conversation at the end of the voting. The process of crystallising may have been interrupted and not rounded off with deep reflection; this might have been due to time limitations or the length of the event. Whilst ideas had been generated, they needed to be tested and piloted in real-life settings (Schnitzler, 2020). Although one dish was later trialled, most ideas lacked implementation plans and did not address systemic barriers. Solutions proposed by participants focused on learning and education opportunities rather than modulating systems structures, which cannot be seen as second-order practice change (Poutiatine, 2009). As a result, evidence of systems awareness or second-order change remains unclear.

The workshop encouraged individual and group reflection, helping participants examine their microsystem and evolving perspectives. While the content was valued, participants showed little initiative to act, possibly due to a lack of support structures like mentoring or coaching. Some participants may have felt implementation was outside their role, limiting follow-through.

The chefs appeared to be taken back to the sensing and presencing space after crystallising Planet Kind dishes and Blue-Sky ideas for sustainable menu transformation – possibly due to the short temporal intervention and the need to close the learning loop with reflection. Schnitzler (2020) concludes in their case study review that these processes require long-term assessment and frameworks of entrepreneurial thinking, neither of which were incorporated within this project. The workshop process developed in this pilot supports the critique of Heller (2019) and Vardaman (2020) that Theory U oversimplifies the process of change as a linear journey. Theory U projects, such as the U-Lab, are usually offered over several weeks and months to reinforce awareness and learning loops. Unlike multi-week Theory U programmes such as U-Lab, this one-day workshop offered limited time for reinforcing learning loops. Data was collected only during and shortly after the event. As such, the data does not support the long-term effects of a one-day workshop. Reflection is central to transformational learning (Potter, 2015), but the lack of long-term follow-up may have missed later insights.

The researchers, therefore, conclude that the workshop appears to access systems thinking and aspects of the Theory U process. Transformational change requires further work within the business to react, redesign, reframe, and crystallise other ways of working. Without a follow-up on the workshop, the solutions proposed by participants and the collection of data days after the workshop’s completion have made it challenging to evaluate the full impact of such an event.

#### 4.2.2 Learning loops for transformational change

Poutiatine (2009) describes nine principles of transformational change. The workshop evidence suggests that some of these principles were achieved, whereas evidence for others is lacking. The first principle, as described by Poutiatine (2009), is that transformational change is not linear but cyclical and iterative. The concept of the workshop acknowledges this cyclical nature through its design, and the reference to a seed planted with the gifting of the Carlin peas made this cyclical process overt for participants. Furthermore, the critique above on whether the Theory U process was effectively followed confirms that the participants returned to presencing and reassessed the required changes needed to shift menus rather than following a linear change process.

On the other hand, the limited evidence of the continuation of the suggested opportunities in the Blue-Sky Thinking activity by the participants could be seen as the lack of individual action, which is also an important aspect of transformational change; it means the individual takes on an active and conscious role in creating change. Similarly, the Blue-Sky Thinking ideas do not suggest second-order changes; many appear to reinforce practices already part of the kitchen’s operations, and thus, there is no clear departure from the way menus are created. The paradigm of control within this process was also not challenged.

The participants may have only started to acknowledge the personal dimensions, such as the impact this change has on their rational, emotional, spiritual, imaginative, somatic and socio-cultural being (Poutiatine, 2009). The ‘good’ food and ‘sustainable’ food exercise aimed to access this personal dimension and given the lively discussion and engagement; the exercise likely accessed it. Transformational learning also requires the application of new practices, which participants were only briefly able to explore when they saw the potential change in GHGE by reformulating the recipe, creating Planet Kind Dishes, and considering Blue-Sky Thinking solutions. The workshop was, therefore, limited in its practical application of proposed solutions and did not involve real-life, applied experience of practice change. This means that the principle of the transformational change process appears incomplete; the lack of experience in actively and consciously applying themselves is also likely a reason why there was no clear indication of loss or letting go (Poutiatine, 2009). That is the vulnerability we experience when testing a new way of doing things. Looking specifically at the proposed solutions from the Planet Kind Dish task, both ‘solutions’ are very similar to the current offer the chefs are working with. The Blue-Sky Thinking exercise included a few more suggestions or solutions that had not been tried and tested by this group of chefs, such as creating a sustainable development team, having an allotment, or delivering guest training; however, none of these ideas were realised in the business. This means the opportunity for critical reflection on the paradigm shift was not created and, more importantly, not embodied, and participants reverted to ‘business as usual’. We argue that without testing those ideas and applying the new thinking, the learning loop required for transformational change remains incomplete. Therefore, the transformation process of the workshop may have started but is incomplete and thus reversible (Poutiatine, 2009). There was also a lack of evidence for a change in participants’ worldviews and questioning one’s integrity within the menu transformation process, further highlighting the limits of transformational change and learning achieved with this workshop.

Whilst the workshop evidence supports certain principles of transformational learning and change, there are many principles for transformational change for which no clear evidence was collected in the workshop or the subsequent reflective interviews. This could be linked to the lack of opportunity for participants to implement solutions proposed in the workshop and to embody and assess the change *in situ*.

### 4.3 Workshop insights and data analysis– menu and food offer transformation

The results and the systematic data analysis demonstrate that rich and contextual data can be gained from such an event. While the participants enjoyed and engaged in all workshop sections, the active and lively conversation, as well as the moment of ‘disorientation’ observed by the facilitators when calculating the GHGE of a chosen recipe, indicate that this is an opportunity to consider for future workshops. This was unexpected and had not been planned as part of the data collection activity. Thus, it was only captured as a finding from the reflective dialogues between the facilitators after the event. This is also a good example of a common thread in PAR literature. There has to be flexibility in the design of activities (Zuber-Skerritt, 2015), and such opportunities enable the iteration of research and action cycles.

Creating a table for ‘GF’ and ‘SF’ to explore recurring themes and narratives inductively worked well. However, many academic studies review the discourse and frames of SF from multiple dimensions. Research on changes in the concept of GF tends to focus on one aspect of people’s perceptions, such as moral or ethical, hedonistic or gustatory, cultural and social frames, or personal histories linked to GF or what people perceive as GF. This makes it challenging to compare these themes with other studies. However, Graf et al. (2019) argued that ‘good’ food is ambiguous and contextual. Thus, broad narratives can come to the fore when people discuss GF, with a reduced risk of confirmation bias from the participants, allowing them to access their inner frames for GF and share those with others. This ambiguity of ‘good’ food narratives is visible in the extracted themes. We believe it creates an access point for participants to become aware and understand the complexity of those frames. ‘Good’ food frames in this study appeared more personal and intrinsic, and various concepts were shared. However, some chefs also shared narratives, which were then iterated for SF; this might be due to the overall workshop title and framing, and thus a confirmation bias, or because these frames are crucial to them.

For the SF frames proposed by the participants, our findings align with the sustainability attributes identified by Reynolds et al. (2022). Chefs in this pilot study framed SF as local (better for farmers and linked to lower food miles), organic (specifically better for human health), plant-based and reduced animal consumption (with an acknowledgement of the complexity and the driver being better taste), zero-waste (including food waste but linked with packaging) and minimal packaging (including the gap between the actual environmental impact of packaging). We potentially observed an extension of the attributes by this group of chefs to link local to farm assurance schemes and improved supply chain transparency, the need for menu flexibility in achieving sustainability, a narrative around alternative animal proteins from invasive species, and the use of food preservation techniques to reduce food waste.

For the chefs’ ecosystem task, a table listing actors chosen by each group in descending order of relevance, along with a review of themes recorded from the generative dialogue after the workshop, allowed for exploring some IT. Further, one of the groups had created a rudimentary causal loop diagram, which surfaced different stakeholders’ interactions in the context of menu creation. This task is rooted in concept maps and fuzzy cognitive mapping, thereby helping to explore the constructs of knowledge and worldviews within a group. The chefs’ ecosystems task provided prompts for the mapping exercise. However, this is a limitation as it can frame and direct the participants’ dialogue.

The follow-up task asked participants to consider what aspects influence the menu and food offer and how they prioritise these. Other scholars, such as Mutlu et al. (2022) and Ottenbacher and Harrington (2007), were used as references for the prompts offered to the chefs in this task. This allowed for intra- and inter-group analysis, as well as the evaluation of evidence from previous studies. ‘Food waste’ and the ‘environmental impact of the food offer’ had not been recorded by Mutlu et al. (2022) and Ottenbacher and Harrington (2007), so exploring the priority ranking of these considerations taken by chefs was not possible in reference to these studies. It could bring awareness to inherent tensions in the menu creation process between customer-related drivers, external and internal business needs, overall acceptability and ingredient considerations. This awareness of system drivers, in turn, may enable chefs to envision transformative food offers by identifying levers and navigating the barriers of others.

The participants created the Planet Kind dish as a dish that would consider those menu creation tensions. These were captured as a recipe on flipcharts, including the sustainable narrative the group used to justify their choice of dish. This allowed for intra-group comparison, and because the groups were continuously working together, there was also an opportunity to compare the menu priorities with their proposed dishes. Both have the potential for inductive and deductive coding of the collected data. Notably, Group A ranked sustainability and food waste as having a lower priority of consideration, and they also opted for a solution that appeared to avoid the complexity of menu tensions, as the dish was built by adding different low-emission foods in reference to the GGDOT cards (Armstrong et al., 2020). When the group was asked whether the dish would fit into the concept of their restaurant and whether guests would purchase it, the response indicated that the assumption was that there would be no competing dishes, and thus, the dish would be successfully implemented in the concept restaurant. However, in most dining contexts, there will be more than one option, and that means these dishes compete with each other, adding layers of complexity which appeared to have been avoided by group A. The composition of Group A consisted of fewer senior chefs than Group B, which could be a confounding factor because the chefs in Group A might be less experienced overall in this task.

The Blue-Sky Thinking task provided data that can be deductively and inductively coded, and the voting system also allowed the ranking of the proposed solutions. Further analysis of the potential for transformative change in the solutions can be conducted by comparing them with the nine principles of transformative learning proposed by Poutiatine (2009). The data from the collective workshop debrief and the participants’ and researchers’ reflective interviews were used to explore DT related to the sentiment and ease of different workshop parts. This allowed for reviewing the appropriateness of those sections as well as exploring any recurring IT from those interviews.

Considering the data collected from the workshops and the review above on how it can be systematically analysed, there is confidence that the workshop artefacts can contribute to new perspectives on the menu creation process and identify the barriers and opportunities that chefs see in shifting to food offers with lower GHGE and reduced food waste.

### 4.4 Study limitations

This is pilot-study evidence; thus, the findings are limited by small sample sizes and the risk of selection bias, as participants are more likely to be those interested in the subject who are more likely to attend a day-long workshop. Participants were paid for their time by the restaurant that employed them. Researchers may consider remunerating participants or having businesses cover the cost of their time as part of their continuous professional development offering for chefs, which could help minimise selection bias.

The small sample size and cross-sectional nature of the workshop mean that the findings are potentially difficult to replicate and may be context-dependent; therefore, gathering data from further workshops is suggested. Exploring the chef ‘s micro food system through a workshop has many potential benefits, but this method is very resource-intensive. For example, only a few chefs can be reached because the groups should not be larger than six for a regenerative dialogue, according to Camilleri et al. (2023).

Similar to any PAR study, the researcher’s involvement can lead to confirmation and selection bias. Thus, project development and delivery need input from diverse stakeholders with flat hierarchies to challenge personal biases.

Collecting data with a workshop could also be perceived as risky because the success depends on this one event, and if any of the planned aspects of the event fall short, the whole data collection would be in jeopardy. Additionally, the outputs and findings of the data collection are difficult to predict, requiring the researcher to be flexible in their data analysis approach and timelines. While the workshop’s content and process are repeatable, they are unlikely to create conditions that are fully coherent with one another.

Lastly, as our evidence suggests, real-world transformational change is difficult to quantify without project follow-up and longer-term engagement with participants and partner organisations.

## 5 Conclusion

The literature review of PAR, Theory U, and transformational learning suggested an opportunity for researchers to unpack the micro-processes of key stakeholders’ decision-making, such as chefs and menu transformation. The creation of a workshop for chefs, which requires them to make sense of the menu-creation process, helped co-create data on menu-creation stakeholders and how various menu considerations are prioritised. This presents an opportunity to map and describe the system’s drivers involved in transforming the menu and food offer.

Using participatory methods delivers rich and contextual data. Despite this, findings are potentially hard to replicate due to the small sample size and specific context of each workshop, including people in the room, participants and facilitators; location and set-up of the workshop; work context of the participants; temporality such as what are dominant societal discourses around the time of the workshop; power dynamics of participants and facilitators.

Using Theory U appears to have the potential to drive awareness-based system change, but transformational learning and change require the application and piloting of proposed solutions, which this workshop did not provide. Thus, not all transformational learning principles have been evidenced and may not have been achieved. However, the content, feasibility and engagement with the workshop were positive. Thus, whilst this workshop could offer an opportunity to engage chefs in menu transformation, further evidence is required, and ideally, a longer-term engagement with wider business team members that is more similar to PAR than PAL.

## Abbreviation

DT: Deductive Themes
IT: Inductive Themes
PAR: Participatory Action Research
PAL: Participatory Action Learning
PALAR: Participatory Action Learning and Action Research
GF: Good Food
SF: Sustainable Food
GHGE: Greenhouse Gas Emissions
RA: Research Assistant
LR: Lead researcher
HaFS: Hospitality and Food Service Sector

## Supplementary Material

The Supplementary material for this article can be found online at: https://www.frontiersin.org/articles/10.3389/fsufs.2025.1641960/full#supplementary-material

Supplementary File

## Figures and Tables

**Table 1:** Post-event feedback summary.

Participant ID	001	002	003	Research assistant	Research lead
Feedback format	In-person interview	Written feedback	In-person interview	In-person interview	Self-recorded
Seniority of participant	Senior chef	Chef de partie (mid-senior)	Junior	N/A	N/A
Thoughts on the workshop in general					
Sentiment	Very positive	Very positive	Very positive	Positive	Positive
Scene setting and ground rules					
Sentiment	Positive	Positive	Ok	Too long-winded	To long-winded
Pre-workshop task					
Sentiment	Very positive	Very positive	Very positive	Very positive	Positive
Researcher presentation					
Sentiment	Positive	Very positive	Positive	Positive	Very positive
Chefs ecosystem activity					
Ease of activity	Easy	Moderate	Moderate	Moderate	Easy
Sentiment	Positive	Positive	Positive	Positive	Positive
Improvement suggestion	None	None	None	Combine with the next task	Individual reflective time after the task.
Menu priorities activity					
Ease of activity	Easy	Easy	Ok	Easy	Ok
Sentiment	Positive	Positive	Positive	Positive	Positive
Improvement suggestion	None	None	None	Use the quadrant which we had prepared to prioritise	Individual reflective time after the task.
Sustainable dish activity					
Ease of activity	Easy	Easy	Ok	Moderate to difficult	Moderate
Sentiment	Positive	Positive	Positive	Ok	Ok
Improvement suggestion	Get chefs to cook the dish.	None	None	Invite the group to consciously summarise what has been explored so far in the workshop to make connections and include resources more effectively.	Give more concise direction to focus the group’s thinking.
Blue-Sky thinking					
Ease of activity	Ok	Easy	Ok	Ok	Ok
Sentiment	Not useful	Positive	Positive	Positive	Positive
Improvement suggestion	Get a wider team involved	None	None		
Was everyone able to contribute freely?					
Sentiment	Positive	Positive	Positive	Positive	Positive
Was it well-paced?					
Sentiment	Positive	Positive	Positive	Ok	Ok
Length of workshop					
Sentiment	Very positive	Very positive	Positive	Positive	Very positive
Suitability of venue					
Sentiment	Very positive	Positive	Very positive	Very positive	Positive
Workshop content suitability					
Sentiment	Positive	Positive	Positive	Positive	OK

**Table 2:** Workshop section summary including purpose and connections with theory U and Bronfenbrenner’s systems dimensions.

Workshop section number	Workshop section	Purpose of the section	Theory U^a^ process stage linked	Bronfenbrenner’s systems dimension^b^ targeted
1.	Welcome and creation of a ‘safe’ environment and ground rules	Connecting as people and balancing differences in power	Creating a safe space	Microsystem – intrinsic values
2.	Good food and sustainable food photo elicitation	The Now- what matters to us now, and how do we work now	Seeing and sensing	Microsystem - intrinsic values and macro-systems frames
3.	Researcher presentation about food waste and GHGE of food	The Challenge to the status quo and now	Seeing and sensing	Meso and Exo-systems frames
4.	Creating Menus - Chefs’ ecosystem - fuzzy cognitive mapping	Is the way we work now acknowledging the challenge, who reinforces the ‘now’ and who might support a change?	Sensing, presencing	Meso-systems frames
5.	Menu onion/ladder - menu priorities - group deliberation	What matters most when writing a menu? How is menu content reinforced by the way we work ‘now’?	Sensing, presencing	Meso-systems frames
6.	Creating a sustainable dish	Be free of the current limitations perceived? What if we shift the priority and order?	Crystallising	Exo-systems frames
7.	Blue-Sky Thinking - backcasting	What else might be needed to allow different menu creation and working methods? Practical steps are possible today.	Crystallising	Exo-systems frames
8.	Reflections and feedback	An invitation to consider how the past hours have shaped your thinking: Where do we go from here?	Sensing	Micro-system and reconnect to intrinsic values and macro-system frames.
9.	Graduation - gifting of Carlin pea sprouts and a certificate	The gratitude of facilitators for trusting the process and planting the seed, as this can only be a start.	Sensing, presencing	Micro-systems frames
10.	Follow-up 1 to 1 reflective interviews or questionnaires	Allowing participants to reflect on the journey and deeper embed the acquired perspective	Sensing, presencing	Micro–system – and reconnect to the intrinsic values and macro-system frames.

a Based on the theory U toolkit (Presencing Institute, 2024).

b Based on Bronfenbrenner’s system theory (Bronfenbrenner, 1995; Bronfenbrenner and Morris, 2007).

**Table 3:** Breakdown of participant outreach and attendance.

Participants	Sex		Area of work in business				Seniority level		
	Female	Male	Pastry	Prep Kitchen	Restaurant	Brasserie	Commis	Chef de Partie	Senior Chef
Invites (total 26)	6 (23%)	20 (77%)	6 (23%)	2 (8%)	8 (31%)	10 (38%)	2 (8%)	9 (35%)	15 (58%)
Attendees (total 8)	4 (50%)	4 (50%)	3 (38%)	0 (0%)	1 (13%)	4 (50%)	2 (25%)	4 (50%)	2 (25%)

**Table 4:** Summary of feasibility and practicality rating generated by LR and RA for each workshop section.

Workshop Section	Participants engaged 1–5 (low-high)	Timekeeping 1–5 (low-high)	Practicality of exercise 1–5 (low-high)	Gained desired research outputs 1–5 (low-high)	Theory U process stage linked 1–5 (achieved - not achieved)	Average Rating (STHV)	Comments
Welcome and creation of a ‘safe’ environment and ground rules	4	2	3	4	*Creating a safe* *space* 5	3.6 (1-4)	Although it was not explicitly used to generate research insights, it helped create an environment of trust and openness.
‘Good’ and ‘sustainable’ food photo-elicitation	5	3	4	5	*Seeing and sensing* 4	4.2 (0.84)	This activity may carry the highest risk of facilitator influence towards the group because facilitators shared their images.
Researcher presentation about food waste and GHGE of food	5	4	5	4	*Seeing and sensing* 5	4.6 (0.55)	It was not explicitly used to generate research insights. Still, an active generative dialogue took place during the session, and the activity assessing GHGE impact surprised some participants.
Creating Menus - Chefs’ ecosystem - fuzzy cognitive mapping	5	2	4	3	*Sensing, presencing* 4	3.5 (1-29)	Although partially successful, ideally, facilitators would collect and prompt stakeholders to map their relationships. There was also not enough time for individuals to reflect, which prevented them from connecting to their ‘inner world’.
Menu onion/ ladder - menu priorities - group deliberation	5	3	5	4	*Sensing, presencing* 4	4.2 (0.84)	It was very effective, but recording details of the deliberation process and the final priorities would enrich the data.
Creating a sustainable dish	3	3	3	3	*Crystallising* 2	2.75 (0.5)	The least effective activity could have been due to having had lunch before. It was a very wide-open task with numerous resources available, which may have led to confusion among the participants.
Blue-sky thinking - backcasting	4	4	5	5	*Crystallimg* 3	4.2 (0.84)	Individual tasks in the session are crucial for guiding participants into a reflective space. The dot voting system worked well.
Reflections and feedback	3	3	4	3	*Sensing* 3	3.2 (0.45)	The debriefing felt rushed; therefore, the data collected may not have been as thorough as it could have been.
Graduation - gifting of Carlin pea sprouts and a certificate	5	5	5	N/A	*Sensing, presencing* 3	4.5 (1)	The graduation was very well received and appeared to be meaningful for the participants, but it did not yield any additional data.
Follow-up 1 to 1 reflective interviews or questionnaires	2	3	4	5	*Sensing, presencing* 4	3.6 (1.14)	Only three of the eight chefs engaged in the reflective interviews. The two in-person interviews felt rushed, but participants appeared to gain from the reflections and build a deeper rapport with the researcher on the subject.

## Data Availability

The original contributions presented in the study are included in the article/Supplementary material, further inquiries can be directed to the corresponding author/s.
