# Supplementary material for: Engaging chefs in sustainable practice using Theory U to address greenhouse gas emissions and food waste
Source: Front Sustain Food Syst. Author manuscript; Available in PMC 2026 May 7. (PMC7619067; doi:10.3389/fsufs.2025.1641960)
Supplement: Supplementary File [file EMS213597-supplement-Supplementary_File.docx]

# Appendix

## Appendix 1 Summary of scoping literature review

Table 5 - Summary of methods used in research with chefs

| Number of studies | Method used |
| --- | --- |
| 11 (25%) | Interviews |
| 10 (23%) | Review of literature, media or peer-reviewed |
| 10 (23%) | Survey |
| 9 (20%) | Mixed methods |
| 2 (5%) | Focus groups |
| 1 (2%) | Case study |
| 1 (2%) | Neuro Imaging |
| 44 |  |

Table 6 - Scoping Literature References

|  | Title | Author | Peer reviewed | Journal name | Publication year | Topic | Positionality of the researcher | Method | Method description |
| --- | --- | --- | --- | --- | --- | --- | --- | --- | --- |
| 1 | What makes a great chef? | (Pratten, 2003a) | Yes | British Food Journal | 2003 | Views of chefs and people in the industry on what it takes to be a great chef | Probably within the sector or at least very close to the business | Case study | Case study: Sources include the comments of top chefs, food writers and others within the industry, as well as lengthy discussions with and observations of those working within the sector |
| 2 | Trainee chefs' experiences of alcohol, tobacco and drug use | (Pidd *et al.*, 2014) | Yes | Journal of Hospitality and Tourism Management | 2014 | Views of trainee chefs on alcohol, tobacco and drug use | Academic | Focus group | Focus group |
| 3 | Menu Engineering in the Restaurant Business: A Study on Kitchen Chefs | (Mutlu *et al.*, 2022) | Yes | Journal of Tourism and Gastronomy Studies | 2022 | Views of chefs on the menu engineering process | Academic | Focus group | Focus group |
| 4 | Sustainable international tourist hotels: the role of the executive chef | (Wan *et al.*, 2017) | Yes | International Journal of Contemporary Hospitality Management | 2017 | Explore the competencies of executive chefs to enhance their development and retention. | Academic | Interviews | Behavioural event interviews |
| 5 | Open kitchens: Customers' influence on chefs' working practices | (Graham *et al.*, 2020) | Yes | Journal of Hospitality and Tourism Management | 2020 | Views of chefs about working in open versus closed kitchens | Chef | Interviews | Interviews |
| 6 | Transforming the Food System through Sustainable Gastronomy - How Chefs Engage with Food Democracy | (Richardson and Fernqvist, 2022) | Yes | Journal of Hospitality and Tourism Management | 2022 | Views of sustainable chefs on food democracy | Activist | Interviews | Interviews |
| 7 | Occupational identity and culture: the case of Michelin-starred chefs | (Cooper *et al.*, 2017) | Yes | Journal of Hospitality and Tourism Management | 2016 | Views of Michelin star chefs about identity and culture | Probably within the sector or at least very close to the business | Interviews | Interviews unstructured |
| 8 | Too hot to handle? An analysis of chefs’ job quality in Australian restaurants | (Belardi *et al.*, 2021) | Yes | Journal of Hospitality and Tourism Management | 2021 | Chefs job quality in AUS restaurants | Academic | Interviews | multiple-case study research design |
| 9 | A preliminary study of chefs’ knowledge and attitude towards nutrition during restaurant’s food handling | (Adhianata *et al.*, 2023) | Yes | Journal of Hospitality and Tourism Management | 2023 | Chefs view on nutrition, their nutrition knowledge | Academic | Interviews | Questionnaire |
| 10 | Resourcefulness of chefs and food waste prevention in fine dining restaurants | (Filimonau *et al.*, 2023) | Yes | Journal of Hospitality and Tourism Management | 2023 | Explores resourcefulness of chefs and food waste prevention | Academic | Interviews | Semi-structured Interviews |
| 11 | The impact of sustainability and leadership on the innovation management of Michelin-starred chefs | (Mrusek *et al.*, 2022) | Yes | Journal of Hospitality and Tourism Management | 2022 | Views of chef on innovation, sustainability and leadership | Academic | Interviews | Semi-structured interviews |
| 12 | Knowledge Transfer in Haute Cuisine: The Relationship between Chefs as an Enabler Factor | (Escalante *et al.*, 2022) | Yes | Journal of Hospitality and Tourism Management | 2022 | Views of chefs on how they share knowledge | Academic | Interviews | Semi-structured interviews - A qualitative methodology is applied based on the analysis of in-depth interviews. |
| 13 | Chefs and occupational culture in a hotel chain: A grid-group analysis | (Cameron, 2001) | Yes | Journal of Hospitality and Tourism Management | 2001 | Identity of chefs/culture | probably within the business or at least very close to the business | Interviews | Semi-structured interviews and grid-group analysis |
| 14 | The innovation development process of Michelin-starred chefs | (Ottenbacher and Harrington, 2007) | Yes | Journal of Hospitality and Tourism Management | 2007 | Views of chefs about their innovation process | Academic | Interviews | Semi-structured interviews with Michelin star chefs |
| 15 | The Parisian Cooks’ Union and Chefs de Cuisine (1880s-1930s) Arch-enemies or Allies? | (Van den Eeckhout, 2017) | Yes | Journal of Hospitality and Tourism Management | 2017 | Historical analysis exploring interactions of kitchen hierarchies and unions in Paris in the 1920s | Academic | Literature review | Historical evidence reviews |
| 16 | Chef's competency as a key element in food tourism success: A literature review | (Mahfud *et al.*, 2019) | Yes | Journal of Hospitality and Tourism Management | 2019 | Grounded theory to review 38 articles | Academic | Literature review | Literature review |
| 17 | National scarce skills within the Professional Chef’s sector in South Africa | (Balkaran and Giampiccoli, 2013) | Yes | Journal of Hospitality and Tourism Management | 2013 | Academic views of the sector | Academic | Literature review | Literature review |
| 18 | Chefs as change-makers from the kitchen: Indigenous knowledge and traditional food as sustainability innovations | (Pereira *et al.*, 2019) | Yes | Journal of Hospitality and Tourism Management | 2019 | Views of chefs from case studies from SA and Mexico | Activist | Literature review | Literature review and case studies |
| 19 | Developing a framework for understanding the impact of deskilling and standardisation on the turnover and attrition of chefs | (Robinson and Barron, 2007) | Yes | Journal of Hospitality and Tourism Management | 2007 | Academic views of the sector | Academic | Literature review | Literature review and development of framework |
| 20 | Examining Chefs’ Social Responsibility (CSR) during the COVID-19 pandemic | (De Guzman *et al.*, 2022) | Yes | Journal of Hospitality and Tourism Management | 2022 | Views and online opinions of chefs | Academic | Literature review | Media Study |
| 21 | A Revolution in an Eggcup? Supermarket Wars, Celebrity Chefs and Ethical Consumption | (Lewis and Huber, 2015) | Yes | Journal of Hospitality and Tourism Management | 2015 | Thematic analysis of media campaigns linked to chefs | Academic | Literature review | Media Study |
| 22 | Credentialed Chefs as Certified Wellness Coaches: Call for Action | (Polak *et al.*, 2015) | Yes | Journal of Hospitality and Tourism Management | 2015 | Views of academics on chefs providing cooking training for the general public | Academic | Literature review | Reflections' Review view of literature and a coaching programme targeting chefs |
| 23 | The changing role of the chef: A dialogue | (McBride and Flore, 2019) | Yes | Journal of Hospitality and Tourism Management | 2019 | Reflections of academics and chefs on how the role of chefs has changed | Academic & Chef | Literature review | Reflective collection of research experiences |
| 24 | The role of the celebrity chef | (Giousmpasoglou *et al.*, 2020) | Yes | Journal of Hospitality and Tourism Management | 2020 | Role of celebrity chefs by proxy | Academics | Literature review | Review of existing research on chefs, deriving from secondary data and academic literature |
| 25 | Bringing sustainable seafood back to the table: exploring chefs’ knowledge, attitudes and practices in Peru | (De La Lama *et al.*, 2020) | Yes | Journal of Hospitality and Tourism Management | 2018 | Views of chefs on sustainable seafood - Knowledge, Attitudes and Practices Framework | Activist | Mixed | Case study: Sources include the comments of top chefs, food writers and others within the industry, as well as lengthy discussions with and observations of those working within the sector |
| 26 | A phenomenological approach to hospitality management research: Chefs’ occupational commitment | (Robinson *et al.*, 2014) | Yes | Journal of Hospitality and Tourism Management | 2014 | Views of chefs and their occupational commitment | Chef | Mixed | Mixed Methods - phenomenological approach sequential mixed method design which incorporates an exploratory quantitative phase preceding the substantive qualitative phase. |
| 27 | Chefs and researchers: Culinary practitioners' views on interaction between gastronomy and sciences | (Fooladi *et al.*, 2019) | Yes | Journal of Hospitality and Tourism Management | 2019 | Views of Food Professionals Working with Researchers | Chefs and academics | Mixed | Mixed methods - quantitative survey and written accounts |
| 28 | The training and retention of chefs | (Pratten, 2003b) | Yes | Journal of Hospitality and Tourism Management | 2003 | Views of chefs and people in the industry on training and retention of chefs | Probably within the business or at least very close to the business | Mixed | Mixed methods, visit to a college, Interviews of 10 restaurants and their staff. |
| 29 | How Michelin-starred chefs are being transformed into social bricoleurs? An online qualitative study of luxury food service during the pandemic crisis | (Batat, 2021) | Yes | Journal of Hospitality and Tourism Management | 2021 | Views of Michelin Chefs on social engagement of chefs during the pandemic | Academic | Mixed | Mixed methods: exploratory qualitative research that used mixed-method, combining online interviews with 12 French Michelin-starred chefs and archival data |
| 30 | Pillars of sustainable food experiences in the luxury gastronomy sector: A qualitative exploration of Michelin-starred chefs’ motivations | (Batat, 2020) | Yes | Journal of Hospitality and Tourism Management | 2020 | Views and attitudes of chefs | Academic/chef | Mixed | Mixed methods: interviews, archival data, and observational notes |
| 31 | “Just trained to be a chef, not a leader”: A study of head chef practices | (Wellton *et al.*, 2019) | Yes | Journal of Hospitality and Tourism Management | 2019 | practices of chefs and their reflections on them | Chef and academic | Mixed | Mixed methods: observation and interviews |
| 32 | The Influence of TV and Celebrity Chefs on Public Attitudes and Behavior Among the English Public | (Caraher *et al.*, 2000) | Yes | Journal of Hospitality and Tourism Management | 2000 | Views/influence of celebrity chefs' assessment of chefs by proxy | Not in the industry by adjacent | Mixed | Mixed methods: Survey, interviews and focus groups |
| 33 | Women chefs’ experience: Kitchen barriers and success factors | (Haddaji *et al.*, 2017) | Yes | Journal of Hospitality and Tourism Management | 2017 | Views of Female chefs about barriers and Opportunities in Kitchens | Academic | Mixed | Semi-structured interviews and focus groups with eight female chefs |
| 34 | Increased cerebellar gray matter volume in head chefs | (Cerasa *et al.*, 2017) | Yes | Journal of Hospitality and Tourism Management | 2017 | Neuroimaging of head chefs to assess brain adaptations resulting from the motor and cognitive demands of working as a chef. | Academic | Neuro image | Neuroimaging |
| 35 | Incivility, satisfaction and turnover intention of tourist hotel chefs Moderating effects of emotional intelligence | (Chen and Wang, 2019) | Yes | Journal of Hospitality and Tourism Management | 2019 | Views on job satisfaction, incivility and turnover intention, emotional intelligence of hotel chefs | Academic | Survey | Structured survey |
| 36 | The price of success: A study on chefs’ subjective well-being, job satisfaction, and human values | (Ariza-Montes *et al.*, 2018) | Yes | Journal of Hospitality and Tourism Management | 2018 | Cooks and chefs’ views - information on the attitudes, beliefs, and behaviours | Academic | Survey | Subset of European-wide survey on attitudes, beliefs, and behaviours |
| 37 | Occupational stress: A case study among chefs and kitchen workers. | (Kohli and Mehta, 2022) | Yes | Journal of Hospitality and Tourism Management | 2022 | Occupational hazards of chefs | Academic | Survey | Survey |
| 38 | An examination of social support and social identity factors and their relationship to certified chefs' burnout | (Kang *et al.*, 2010) | Yes | Journal of Hospitality and Tourism Management | 2010 | Views of chefs on social support and social identity | Academic | Survey | Survey |
| 39 | A comparative exploration of celebrity chef influence on millennials | (Cifelli *et al.*, 2020) | Yes | Journal of Hospitality and Tourism Management | 2020 | Views of Millennials on Celebrity Chefs | Academic - Advocacy | Survey | Survey |
| 40 | The Differences Between ACF (American Culinary Federation) Professional Chefs and Chef Educators: Concern for Task Versus Concern for People | (Swift *et al.*, 2019) | Yes | Journal of Hospitality and Tourism Management | 2019 | Views of members of the ACF on priorities between tasks and concern for people | Academic | Survey | Survey |
| 41 | Chefs’competencies: a stakeholder’s perspective | (Marinakou and Giousmpasoglou, 2022) | Yes | Journal of Hospitality and Tourism Management | 2022 | Views of chefs, trainees and chef educators on required competencies | Academic | Survey | Survey |
| 42 | Chefs' Opinions About Reducing the Calorie Content of Menu Items in Restaurants | (Obbagy *et al.*, 2011) | Yes | Journal of Hospitality and Tourism Management | 2011 | Views/opinions of chefs on 'healthy' food | Not in the industry by adjacent | Survey | Survey |
| 43 | Chefs’ Opinions of Restaurant Portion Sizes | (Condrasky *et al.*, 2007) | Yes | Journal of Hospitality and Tourism Management | 2007 | Views/opinions of chefs on 'healthy' food | Not in the industry by adjacent | Survey | Survey |
| 44 | Attitudes and beliefs about how chefs can promote nutrition and sustainable food systems among students at a US culinary school | (Bertoldo *et al.*, 2022) | Yes | Journal of Hospitality and Tourism Management | 2022 | Attitudes and perceptions of culinary students about nutrition and sustainability as part of their roles, responsibilities and future work as chefs | Academic | Survey | Survey |

## Appendix 2 Ethical Considerations

Table 7 - Ethical considerations and preventative action taken

| Ethical consideration | Preventive Action |
| --- | --- |
| Benefit for the participants in giving their time – including potential remuneration for the time | The collaboration business agreed to pay the chefs in full for the time of the workshop. |
| Workshop accessibility | A location close to the usual working place was selected, which was easily accessible via public transport for all participants. Additionally, the LR provided support with wayfinding. |
| Selection criteria of participants – to create a meaningful insight | A selection criterion was agreed on before the start of the recruitment. |
| Safe and supportive work environment for diverse people (culturally – ethnic diversity, mental - neurodiversity and physical – diversity in abilities) | The participants and researchers created joint ground rules for the workshop day. |
| The role of the employer, employee and researcher and their respective boundaries | All participants received an information sheet, a consent form, and a follow-up email from the LR after the workshop, providing them with an opportunity to fully understand the context of the research and the workshop, ask questions, and withdraw from the workshop and the research at any time. |
| Support required in the lead-up, during and after the workshop for participants and facilitators | The contact details of the LR were shared before and after the workshop to facilitate follow-up at suitable times for participants. |
| Location and hospitality offer | A partner university venue was selected to ensure the implementation of sufficient and suitable health and safety measures, as well as the required public liability insurance. The university's contracted caterer was also used for the same reasons. |
| Positionality of facilitators | The LR developed a selection framework for the RA, which is presented in Appendix 3. |
| Power hierarchies of participants and facilitators | The LR and RA had previous experience and training delivering workshops and actively observed the group. The aim was to reduce the hierarchies typically followed by this group of chefs. The group was also divided into subgroups to further disrupt standard hierarchies and power structures. The facilitators contributed to the good food and sustainable food exercises intending to level the group hierarchy. |
| The researcher had previously worked with all the participants and was familiar with the premises where the chefs worked; however, the RA was not familiar with the business or the participants. | The RA conducted a site visit prior to the workshop to observe the context in which the participants work and gain an understanding of the work culture. The LR and the RA further discussed their positionality to ensure that there was trust, support and the possibility to challenge each other during the workshop if needed. |
| Ethical sharing of data and ensuring anonymity | Participants consented to the capture of their data after having received all relevant information. Participants had the right to withdraw their data at any point within the ethical approval period. Any pictures taken during the workshop did not contain people's faces to maintain anonymity. Data collected during the workshop did not contain names or was anonymised. Data collected during the workshop was shared with the participants before use for research reports, and participants received regular updates on the research progress. |

Ethics application process

The project was presented to the Regional Hospitality Manager, the General Manager, the HR Business Partner, the Executive Chef, and, finally, the senior chef team of the partner business to gain their consent for the delivery of the workshop. During the presentation, feedback was gathered verbally and used to inform the design of the workshop. A permission letter from the business was received and added to the ethics application.

The ethics application contained the following documents: a consent form (including consent for photos and recording), a participant information sheet, a focus group schedule, a general risk assessment, a semi-structured interview guide for follow-up after the workshop, and an email invitation for participants. Ethics approval (44491-LR-Aug/2023- 46866-1) was received at the end of August 2023.

For the event venue, a general emergency evacuation procedure was established, and a small-scale risk assessment was conducted, including risks associated with the provision of refreshments. As the event venue and their caterer were at an associated university, health and safety considerations within the ethics were fulfilled.

Participant selection / Inclusion criteria

Participants had to be at least 18 years old, currently employed as chefs in the partner business, and have worked as chefs for a minimum of 2 years.

Researcher Assistant (RA) Recruitment and Responsibilities

A detailed description of the RA recruitment, selection and responsibilities in the broader research and the workshop can be found in Appendix 3.

## Appendix 3 Workshop development

### Methodological development

PAR is grounded in a research paradigm which acknowledges the agency of people involved in a research process (Macdonald, 2012) and recognises that the interaction between researchers and participants has the potential to drive action and change. As a research approach that involves researchers becoming part of a cyclical process of transformation, PAR advocates that research should be more than gathering evidence; it should also help drive societal change. PAR aims for researchers to obtain knowledge by actively participating in and supporting change processes (Walter, 199). This approach should be collaborative and equal, meaning it is necessary to balance power dynamics and structures withn these processes. Thus, PAR is not confined to specific techniques and methods but encompasses a particular research approach (Cornwall and Jewkes, 1995).

PAL is the collective learning process in groups within PAR settings, which typically aims to address a specific challenge or problem. Participatory methods encompass a range of methods and techniques used in PAR and PAL. These methods employ techniques and tools that engage people actively in research, learning, or decision-making processes, enabling them to make sense of a problem (De Jaegher and Di Paolo, 2007). Tools are flexible and usually respond to the needs of a group and a problem. They can involve focus groups, stakeholder mapping, photo-elicitation and storytelling (Frasso *et al.*, 2018). These methods are applied in repeated cycles of action and learning.

PAL has been linked to experiential and transformational learning theories (Percy, 2005; Schnitzler, 2020; Zuber-Skerritt, 2015) which contend that these methods allow adults to access tacit knowledge in first-order learning experiences and transform it into second-order learning experiences. This means that incomplete tacit knowledge is confronted with unexpected or surprising outcomes that require reassessment of beliefs and knowledge. This is achieved in the process of reflection after the experience. For transformational learning to occur, critical reflection and dialogue play a key role in sense-making. The transformational learning theory builds on the experiential learning theory (in Azar et al., 2020; Percy, 2005) and suggests that leaps in knowledge or changes in an individual’s perspective require critical reflection on the dissonance between previous knowledge and the learning experience, which can be created in workshops. Schnitzler (2020) refers to four distinct levels of transformational learning linked to Theory U: reacting, redesigning, reframing and presencing.

An essential difference between interviews, surveys and PAR methods can be seen in the researcher's positionality when entering the discourse studied with the ambition of social change (Macdonald, 2012). The facilitator invites participants to explore topics through dialogue and conversation in focus groups, often drawing on their prior knowledge and experience. Participatory methods, on the other hand, introduce concepts and ideas and invite participants to an embodied co-creation process for relational sense-making (De Jaegher and Di Paolo, 2007), which often requires participants to use paper, pen, and images to visualise their opinions. At the end of most workshops, a reflective phase invites the participants to consider the images and concepts they created as a group. Therefore, the analysis focuses on the action of sense-making—the creation of meaning in new contexts—and how it may have evolved. With participatory methods, participants are actively making sense of the subject, which can be linked to adult learning theories such as Fischer's dynamic skills theory (2009), experiential learning theory first proposed by Kolb (in Azar et al., 2020; Ochago et al., 2024), Mezirow’s transformational learning theory (Carter and Nicolaides, 2023) and systems change-facilitation (Birney *et al.*, 2025; Scharmer, 2016) whereas focus groups tend to emphasise the reflection and dialogue of participants' lived experiences rather than the co-created discourse. Qualitative methods of inquiry, such as PAR workshops, can enrich the understanding of system dynamics (Birney *et al.*, 2025; Luna-Reyes and Andersen, 2003) and knowledge creation (van Dijk, 2024).

### Research assistant (RA) recruitment and responsibilities

A RA was recruited for this phase of the wider project. The primary responsibility of the RA was to support the delivery of the workshop, as there were many logistical tasks to be completed. One of the potential drawbacks of participatory methods is that the researcher's objectivity is limited due to social interaction with the participants. By involving a second observer and practitioner, a second perspective on the same event is introduced. This suggests that the criteria for this person needed consideration before the start of the project. The aim was for the support person to have a different research perspective and relationship to the participant group. Equally, that person needed to have sufficient prior knowledge of the theoretical research context to feel competent and comfortable leading an inquiry into the subject at hand.

Working with an RA throughout this phase of the project would provide benefits such as:

- Ensuring the quality of the workshop experience is considered.
- Another critical eye on the research process.
- Assistance with data collection is needed, as a large amount of data will be generated during the workshop.
- Reduction of selection bias.
- Elevating and combining the skills required in this process.
- Opportunity for peer-to-peer learning.
- Making this a transdisciplinary learning experience for all involved parties
- Opportunity to improve the people management and teamwork skills of both researchers.
- Improving the safety of the participants and the lead researcher.

Since the RA would take an active role as a peer-to-peer advisor, a framework for selecting the assistant was created (Mercer-Mapstone *et al.*, 2017).

To ensure that potential benefits are realised, it was essential to communicate the expectations of the RA to the ethics application and the research supervisors. So, these were incorporated into the written recruitment and work framework. The LR explicitly stated how the collaborative work was intended to commence. It included the capabilities the RA should have and the necessary personal profile, similar to the stakeholder and participant choices for the workshop, which had an inclusion criterion.

The LR set the following inclusion criteria for the RA:

- - Must have an undergraduate degree – preferred social sciences background due to the nature of the project
  - Must have experience in thematic analysis and knowledge of participatory research methods, and experience with those would be beneficial.
  - Must have good people skills and a desire to work in a team setting
  - It must be someone who has a different entry point of the food system than the LR, so the positionality of the LR can be challenged during the peer-to-peer review processes
  - Must have food systems literacy and an interest in food systems work
  - Must be interested in interdisciplinary and transdisciplinary research discourse
  - Must have worked with diverse groups of people, especially non-academics
  - Must be able to travel to the workshop site
  - Minimum availability for the project work package A and B, with the possibility to opt out of work package C.

To recruit the RA, the LR utilised the network of the UK Food Systems Centre for Doctoral Training, the School of Systems Change, and the contacts of the project supervisors. A breakdown of the project was drafted, including specific tasks, expectations, and allocated time commitments. This was shared with potential candidates, and in-person conversations and selection were carried out. A consultancy role was created as part of this process.

### Workshop co-creation

After recruiting a suitable RA, a pre-workshop co-creation process began, led by the LR through regular, mostly virtual meetings with the RA. During these meetings, the LR proposed the structure and content for the workshop. It was then further developed iteratively between both researchers, the project supervisors and the executive chef of the pilot business. The aim was to minimise the positionality bias of the lead researcher, who still works in the field of research, and to aid the design of the workshop, including suggested methods and terminologies, timelines, etc. This iterative development took place between July and mid-Sep 2023. Records of adjustments were kept for the follow-up workshops and the subsequent interim report of the project.

The LR attended several industry workshops targeting chefs hosted by Climate Smart Chefs, The Chef's Manifesto, Guardians of Grub, Great Taste Zero Waste, the Sustainable Restaurant Association, and culinary colleges to observe how chefs engaged with these events. Where possible, reflective summaries were written, and official records of the events were kept. This further supported the thinking around the workshop design.

### Selection of workshop themes and structure

The structure was designed with participants in mind, enabling them to navigate the U process within the workshop context, drawing on transformational and experiential learning theory, as well as dynamic skills theory. The methods used were inspired by participatory methods used in food systems research previously, and reference was made to the stakeholder interview themes (Zick et al., 2025); current systems change facilitation practices; photo elicitation participatory practices, food systems literacy research informed by IFSTAL (Pope *et al.*, 2021), systems change facilitation practices acquired through the Spark course of the School of Systems Change (School of Systems Change, 2025) as well as to Theory U (Presencing Institute, 2024).

## Appendix 4 Workshop Overview

Table 8 - Detailed workshop outline

| Time | Task | Assigned to | Reasoning |
| --- | --- | --- | --- |
| 9:00 | Both Facilitators arrive  Set up flipcharts, chairs, tables, stationery, a clock, laptops, and a microphone. | Both Facilitators | Prepare and organise the space for the smooth running of a diverse set of activities. |
| Creating a safe space | | | |
| 9:15 | Coffee, Tea & Pastries set-up | Catering team |  |
| 9:30 | Participants arrive and write their name badges.  Re-share the participant information sheet and ask them to sign the consent form to take part. | Both Facilitators | Create a space that fosters the sharing of experiences and knowledge on common ground. |
| 10:05 | Health and Safety Briefing & Consent Form | LR | A space that builds connection and trust, which participants can take back to their workplace. |
| 10:10 | Good Food Session | RA moderates,  LR to capture information on a flipchart. | An opportunity to introduce ourselves and hear about the different ways good food can be interpreted, reinforcing that we all have different ways of seeing the world. Neither view is inherently wrong but merely influenced by our contexts, and there is value in this diversity.  Facilitators to highlight commonalities and differences, and how these might support each other |
| 10:40 | Setting Ground Rules – LR to moderate, RA to support. | LR moderates,  RA to capture output. | This is to ensure participants realise they have autonomy and can actively shape the workshop (some may not have been in situations like this before). So, facilitators will support this by offering a selection of possible ground rules picked up randomly from a hat. The group can agree on whether they wish for that rule to be applied for this workshop, and blanks were added for the group to create their own rules.  Facilitators need to reinforce the agreed-upon ground rules throughout the workshop. If, for any reason, a ground rule becomes problematic, the group should seek consensus on how to address it. |
| 10:50 | Coffee, Tea, Snacks – 5 min break |  |  |
| Making sense of sustainable food | | | |
| 10:55 | Sustainable Food Session | RA moderates,  LR to capture information on a flipchart. | This is for the facilitators to pick up the knowledge of sustainable food frames of the group and to allow peer-to-peer learning before the facilitators bring some 'new' learning into the group to support the planned group work. |
| 11:20 | GHGE, carbon cycle of cow and food waste presentation to end with mini GHGE recipe assessment. | LR mostly, but RA does the carbon cycle of the cow. | The stakeholder interviews have highlighted that the GHGE of food is a problematic concept. While many chefs and other stakeholders appear to have a basic understanding of the GHGE impact of foods, it is often oversimplified, i.e., local is better than global, and meat is considered better than plants.  Facilitators aim to explain why considering GHGE matters to chefs, give practice-based examples, share the complexity of the concept and how food waste and GHGE link, and offer trusted resources for further exploration, as the topic is too large to be covered in 20 minutes.  Some of the resources will also be made available to the chefs in the room during their group work. |
| 11:40 | Coffee and tea break |  |  |
| Mapping out the current system | | | |
|  | Group Work |  | Ensure the group size is balanced and does not exceed 6 to facilitate effective communication and collaboration. When setting up the group, we ensured a mix of levels of experience, gender, and representation from the different kitchen teams. |
| 11:45 | Chef’s Eco-System – 30 min group work & 15 min reflection and discussion. | LR to explain the group task.  LR and RA to support group A and group B, both to capture output jointly with the group on a flipchart. | This is for the chefs to consider who influences their decisions when deciding which foods to put on the menu. Some participants may not have considered this explicitly, but it presents an opportunity for chefs to share their expertise with facilitators and engage in peer-to-peer learning. By raising awareness of how other actors influence their work, they can develop strategies to shape these relationships actively.  For the facilitators, it will enable them to understand some of the key synergies between ecosystem actors and explore barriers and opportunities that may help in other group work, such as when the chefs decide on menu priorities and come up with a planet-friendly dish. The conversation may also highlight how far food waste and GHGE are perceived to be driven by certain actors in this ecosystem rather than by others. |
| 12:15 | Lunch delivered/set up | Catering team |  |
| 12:25 | Lunch break 50 min |  |  |
| Exploring the dynamics of the current system | | | |
| 13:15 | The Menu Hierarchy – 20 min group work & 15 min reflection and discussion | LR to explain the group task.  LR and RA to support group A and group B, both to capture output jointly with the group on a flipchart. | The aim of this is to consider the wider food offer and how a menu is composed versus a single dish. Likely, some of the chefs have not overtly considered the various decision dimensions that lead to the creation of a menu. This means there is an opportunity for peer-to-peer learning among the chefs, helping to build more alignment within the team and also enhancing each participant's ability to understand the complexity of food offer creation and its impact on food waste and GHGE. |
| 13:45 | Coffee and tea break |  |  |
| Imagining different futures | | | |
| 13:50 | Planet-Kind dishes – 30 min group work & 15 min reflection and discussion | LR to explain the group task.  LR and RA to support group A and group B, both to capture output jointly with the group on a flipchart. | This allows the chefs to share their passion for food and apply some of what they have learned during the earlier part of the day. This group work is likely to spark a dialogue about the various considerations one may take when discussing a planet-friendly meal. This may not imply that the dish has low food waste or particularly low GHGE; however, the process of building an argument or narrative around the planet-friendly theme may trigger their curiosity and creativity and could help them take the seeds of ideas back to work.  The facilitators should actively encourage dialogue between the group and probe into the different stakeholders and menu dimensions that have been brought about in the previous two group works, thereby encouraging self-reflection among the chefs. |
| 14:50 | Coffee, Tea, Snacks – 5 min break |  |  |
| 14:55 | Blue-Sky Thinking / Action Planning  Participants have 20 minutes to write as many post-its as possible for all timelines  20min adding post-its to the different flipcharts  5 min voting on the most popular joint actions with dot stickers | RA to moderate and LR to capture evidence on a flipchart. | This part of the workshop is designed for chefs to actively think about how they can create a work environment that is more supportive for them. To think about what they might need to feel in a position to cook 5 or 10 years from now, and how they might prepare for the potential future ahead.  If possible, actions, ambitions, and goals are divided into 1 year, 5 years, and 10 years from now.  The facilitators should encourage each idea to be developed into a SMART goal, making the potential application more feasible and realistic. There is an opportunity for the chefs to include personal goals and choose whether they wish to share these with the group or use this exercise to consider how they can make their personal goals a smart goal. |
| 15:55 | Coffee and tea break |  |  |
| 16:00 | Wish list for facilitators – how to share what was learned, created, and what support is needed. | LR to explain the group task.  LR captures responses on a flipchart. | This part of the workshop is for the facilitators to explore how the workshop's learnings should be shared, what additional resources could be provided, and whether there is something on the list that the facilitators could help with. |
| 16:15 | Debrief what worked – Open Circle | LR to explain the group task  LR captures responses on a flipchart | An opportunity to share what was of value to each person, the facilitators should use this to encourage each person to seek out more of that and actively create contexts where these positive aspects can be a more frequent experience as for any criticism, it is essential for the facilitators to actively explore the criticism so in becomes constructive criticism which can be actioned. |
| 16:50 | Handing out completion certificates and planted seeds, participants depart. | LR and RA joint moderation and congratulations. |  |

## Appendix 5 Workshop equipment list

- Timer/clock
- GHGE - GGDOT flashcards (Armstrong *et al.*, 2020)
- Prepared flipchart for good food, and sustainable food, chefs' ecosystem game and menu hierarchy, planet-friendly dish
- Print/cut-outs of prompts for the above and ground rules
- Flipcharts, post-its, Sharpies, fidget spinners, note paper, name badges
- Laptop for photos and presentations, as well as for music during breaks
- Recording device
- Camera to collect evidence

## Appendix 6 Artefacts

### Proposed Artefact analysis

Each of the workshop sections will produce workshop artefacts, including flipchart images and written content, which serve as records of collective sense-making. We argue that these can be analysed with reference to other research. Below, we suggest a systematic analysis for each of the workshop sections.

1. Creating a table for ‘good food’ and ‘sustainable food’ to explore reoccurring themes and narratives inductively. Using methods from group photo elicitation studies such as those applied by Diaconeasa et al., 2022; and Mapes and Ross, 2022 and comparing and contrasting findings with studies like Graf et al., 2019; Reynolds et al., 2022.
2. For the chefs’ ecosystem task, a table listing and comparing actors chosen by each group in descending order of relevance can be created. This table can then be further reviewed to record themes from generative dialogue after the workshop, allowing IT to be explored. This task is rooted in concept maps and fuzzy cognitive mapping (Henly-Shepard *et al.*, 2015), thereby helping to explore the constructs of knowledge and worldviews within a group.
3. For the menu priorities tasks, two academic articles (Mutlu *et al.*, 2022; Ottenbacher and Harrington, 2007) were used to create prompts for the workshop activities. Thus, DT will be explored and compared with those presented in the articles.
4. The creation of a sustainable dish section will enable the comparison of data from both groups, allowing for an examination of the framing of suggested dishes and the approach each group took. Any recurring or divergent themes and concepts, as well as potentially missing themes related to sustainable food, were identified by the participant earlier in the workshop and those found in this rapid review by Reynolds *et al*. (2022). Furthermore, we can compare and contrast the themes with those presented by the participants earlier in the workshop.
5. The Blue-Sky Thinking compares the priorities selected by participants for future action with the narratives and themes explored earlier in the workshop. It includes a voting system for the group. This part of the workshop is based on research on participatory backcasting for sustainable futures (Remans *et al.*, 2024).
6. The data collected from the group debrief at the end of the workshop, the workshop follow-up participant questionnaire and the reflective interview with the RA can be thematically coded and used as evidence for the overall feasibility, practicality and effect on transformational learning.

### Workshop artefacts


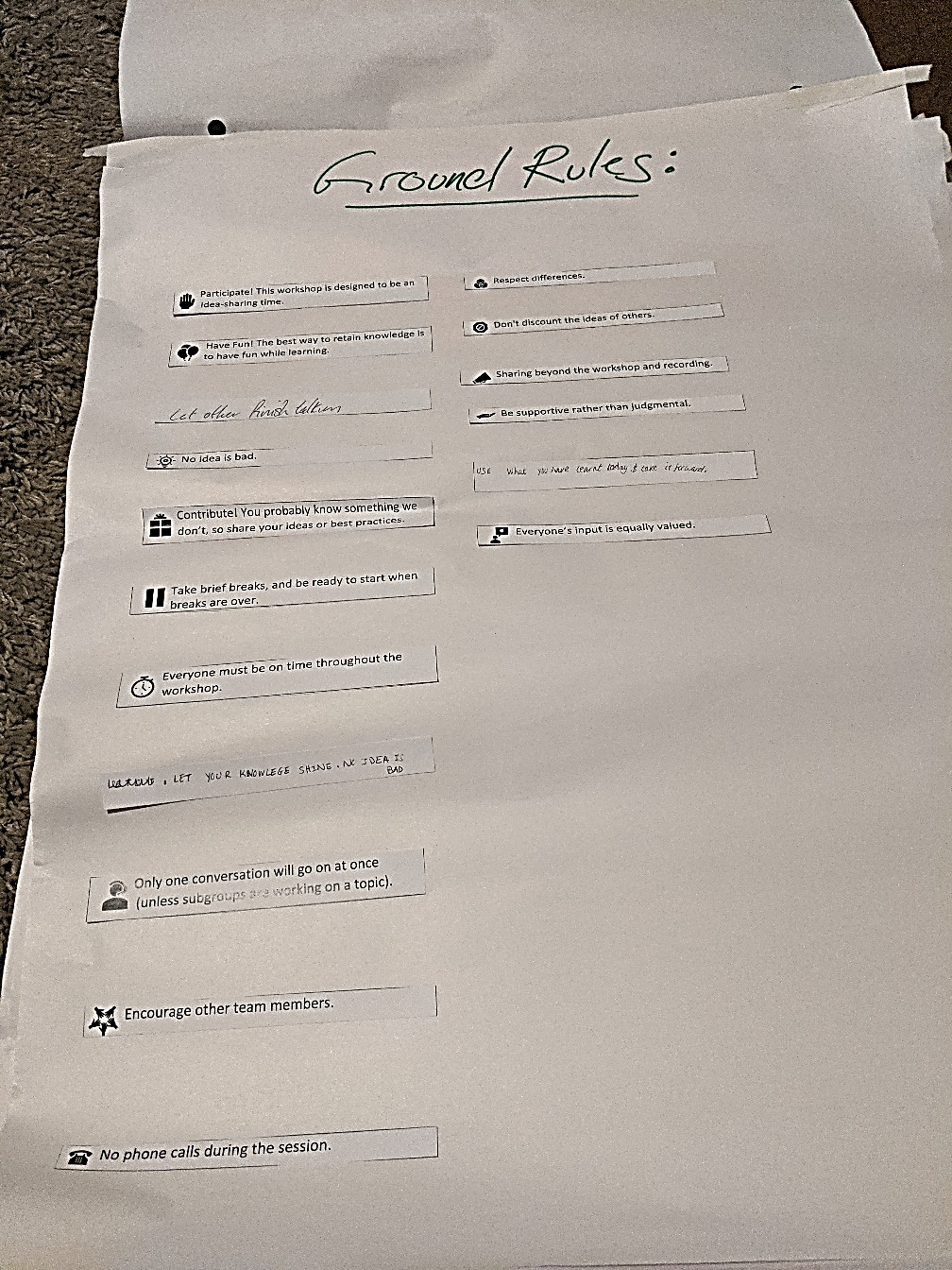


Workshop Artefact 1 – Flipchart of agreed ground rules for the event

Alt Text: A poster with several printed cut-outs titled “Ground Rules” as well as handwritten notes.

| **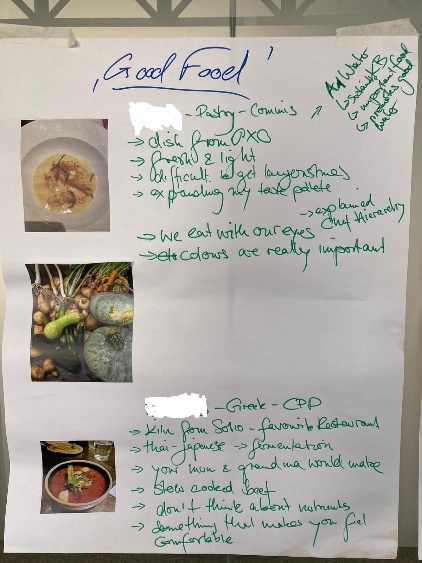** | **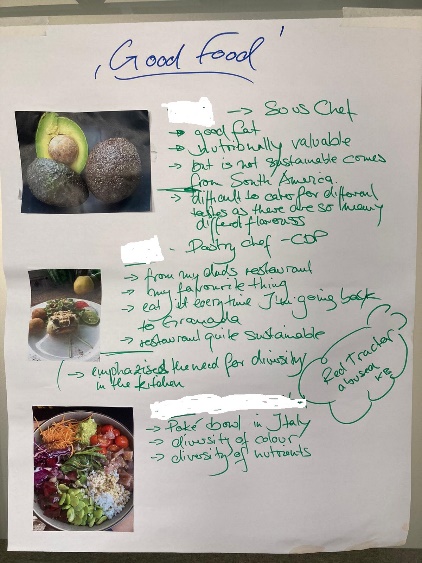** | **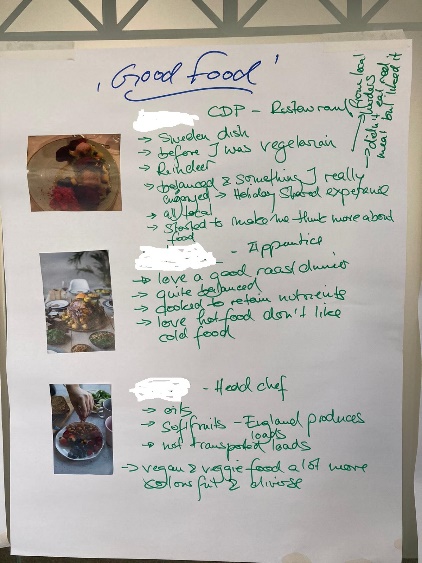** |
| --- | --- | --- |
| Workshop Artefact 2 – Flipchart 1 of Good Food activity | Workshop Artefact 3 - Flipchart 2 of Good Food activity | Workshop Artefact 4 - Flipchart 3 of Good Food activity |
| Alt Text: "Good Food" poster with sections on a seafood and a beef dish, clustered by chef position, including handwritten notes and relevant images. | Alt Text: "Good Food" poster with sections on a salad bowl, crab dish, avocados and clustered by chef positions, including handwritten notes and relevant images. | Alt Text: "Good Food" poster with sections on a Sunday roast, venison dish, mixed berries and clustered by chef positions, including handwritten notes and relevant images |

| 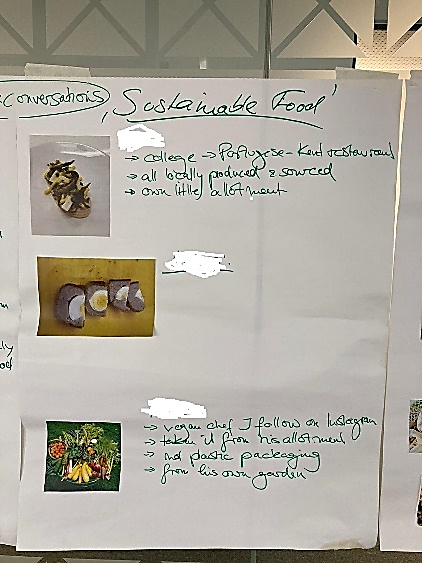 | 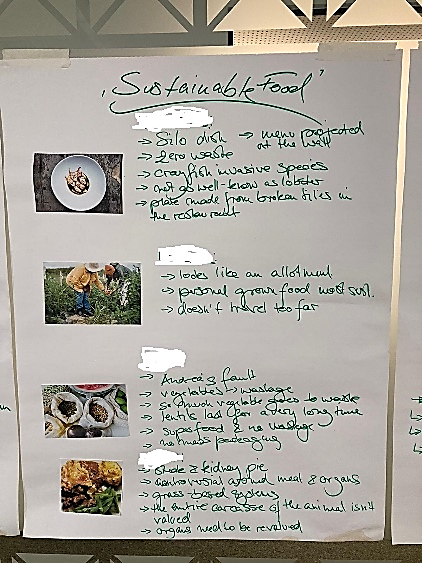 | 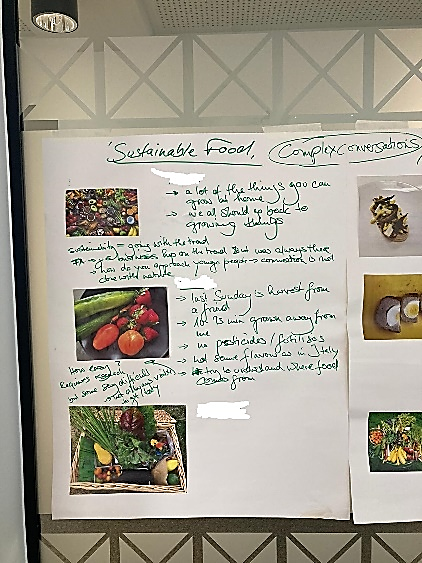 |
| --- | --- | --- |
| Workshop Artefact 5 - Flipchart 1 of Sustainable Food activity | Workshop Artefact 6 - Flipchart 2 of Sustainable Food activity | Workshop Artefact 7 - Flipchart 3 of Sustainable Food activity |
| Alt Text: "Sustainable Food" poster with sections on a locally produced dish, Scotch egg, no-packaging Instagram picture, including handwritten notes and relevant image. | Alt Text: "Sustainable Food" poster with sections on a zero-waste restaurant, an allotment, and a kidney pie picture, including handwritten notes and relevant images. | Alt Text: "Sustainable Food" poster with sections of various fruits, vegetables, grains grown organically, including handwritten notes and relevant images |

Table 9 - 'Good' Food and 'Sustainable' Food Thematic Analysis Summary

| Person identifier | Type of food - GF | GF Own Picture | GF narratives | GF Additional comments | Type of food - SF | SF narratives | SF Own Picture | SF Additional comments |
| --- | --- | --- | --- | --- | --- | --- | --- | --- |
| Participant 1 | Fine dining restaurant soup containing langoustines | Y | fresh and light | Another chef added that langoustines come from UK waters, an important food for the UK | College restaurant, Portuguese dish with ingredients from Kent | Vegetarian | Y | N/A |
| Participant 1 | Fine dining restaurant soup containing langoustines | Y | Langoustines hard to buy in the supermarket - a treat | buying from the right fishers supports economics/communities to take care of water/ecosystem | College restaurant, Portuguese dish with ingredients from Kent | Grown on allotment | Y | N/A |
| Participant 1 | Fine dining restaurant soup containing langoustines | Y | expanding my taste palette | N/A | College restaurant, Portuguese dish with ingredients from Kent | Locally produced and sourced | Y | N/A |
| Participant 1 | Fine dining restaurant soup containing langoustines | Y | visually pleasing - we eat with our eyes | N/A | X | X | X | N/A |
| Participant 1 | Fine dining restaurant soup containing langoustines | Y | food colours important | N/A | X | X | X | N/A |
| Participant 2 | Restaurant dish - slow-cooked beef dish in a sauce from a fine dining restaurant | Y | London-based restaurant | N/A | Fine dining restaurant crayfish on a puree | From a zero-waste restaurant | Y | N/A |
| Participant 2 | Restaurant dish - slow-cooked beef dish in a sauce from a fine dining restaurant | Y | the chefs' favourite restaurant | N/A | Fine dining restaurant crayfish on a puree | Crayfish invasive species in London | Y | N/A |
| Participant 2 | Restaurant dish - slow-cooked beef dish in a sauce from a fine dining restaurant | Y | Thai-Japanese inspired | N/A | Fine dining restaurant crayfish on a puree | Lesser known and used than Lobster | Y | N/A |
| Participant 2 | Restaurant dish - slow-cooked beef dish in a sauce from a fine dining restaurant | Y | Fermented foods | N/A | Fine dining restaurant crayfish on a puree | Menu projected to the wall to save paper | Y | N/A |
| Participant 2 | Restaurant dish - slow-cooked beef dish in a sauce from a fine dining restaurant | Y | Food that makes you feel good | N/A | Fine dining restaurant crayfish on a puree | plates made from broken ones in the restaurant | Y | N/A |
| Participant 2 | Restaurant dish - slow-cooked beef dish in a sauce from a fine dining restaurant | Y | Food that your grandma or mum would make | N/A | X | X | X | N/A |
| Participant 2 | Restaurant dish - slow-cooked beef dish in a sauce from a fine dining restaurant | Y | For good food, I don't think about nutrients | N/A | X | X | X | N/A |
| Participant 3 | Avocados | Y | Good fat | Difficult to cater for many tastes as the chef | Bowl of fruit and vegetables | Last Sundays, harvest from a friend | Y | Sustainability appears to equal going with the current trend |
| Participant 3 | Avocados | Y | Nutritionally valuable - superfood/healthy | N/A | Bowl of fruit and vegetables | 10-15 min grown away from me | Y | Businesses seem to feature this 'trend' more at the moment, but this has always been there. |
| Participant 3 | Avocados | Y | Not sustainable as from South America | N/A | Bowl of fruit and vegetables | no pesticides or fertilisers = organic | Y | How do you approach young people who lack a connection with nature |
| Participant 3 | X | X | X | N/A | Bowl of fruit and vegetables | got flavour as in Italy | Y | N/A |
| Participant 3 | X | X | X | N/A | Bowl of fruit and vegetables | trying to understand where food comes from, but that isn't always easy; here it is | Y | N/A |
| Participant 4 | Restaurant-dressed crab dish with croquettes, salad leaves, lime, cucumber and tomato slices | Y | A dish from chefs' dads' restaurant | The chef emphasized the need for cultural diversity for good food | Table with nuts, seeds, fruits and vegetables | Many plants you could grow at home | Y | N/A |
| Participant 4 | Restaurant-dressed crab dish with croquettes, salad leaves, lime, cucumber and tomato slices | Y | Favourite dish | Another chef raised concerns about the abuse of the Red Tractor certification, which is linked to what is considered sustainable. | Table with nuts, seeds, fruits and vegetables | We should all go back to growing things ourselves | Y | N/A |
| Participant 4 | Restaurant-dressed crab dish with croquettes, salad leaves, lime, cucumber and tomato slices | Y | Dish eaten every time the chef goes home to Granada | N/A | X | X | X | N/A |
| Participant 4 | Restaurant-dressed crab dish with croquettes, salad leaves, lime, cucumber and tomato slices | Y | Dish is quite sustainable for Granada | N/A | X | X | X | N/A |
| Participant 5 | Restaurant dish reindeer with potatoes and vegetables | Y | A dish from a Swedish restaurant | N/A | Table of various vegetables in a garden | Vegan chef on social media | Y | N/A |
| Participant 5 | Restaurant dish reindeer with potatoes and vegetables | Y | Dish before becoming vegetarian | N/A | Table of various vegetables in a garden | Vegetables from his allotment | Y | N/A |
| Participant 5 | Restaurant dish reindeer with potatoes and vegetables | Y | The dish felt balanced; I didn't eat red meat but liked it | N/A | Table of various vegetables in a garden | No plastic packaging | Y | N/A |
| Participant 5 | Restaurant dish reindeer with potatoes and vegetables | Y | I was on a holiday shared with people I care about | N/A | Table of various vegetables in a garden | Homegrown | Y | N/A |
| Participant 5 | Restaurant dish reindeer with potatoes and vegetables | Y | All ingredients were locally sourced from local herders | N/A | X | X | X | N/A |
| Participant 5 | Restaurant dish reindeer with potatoes and vegetables | Y | It made me think more about food | N/A | X | X | X | N/A |
| Participant 6 | Family table with a roast turkey, sharing side dishes, wine glasses | N | Love a good Sunday Roast | N/A | Image of two people growing plants in the field | It looked like an allotment | N | N/A |
| Participant 6 | Family table with a roast turkey, sharing side dishes, wine glasses | N | Quite balanced meal | N/A | Image of two people growing plants in the field | Home-grown = more sustainable | N | N/A |
| Participant 6 | Family table with a roast turkey, sharing side dishes, wine glasses | N | Cooked to retain nutrients | N/A | Image of two people growing plants in the field | Don't travel too far | N | N/A |
| Participant 6 | Family table with a roast turkey, sharing side dishes, wine glasses | N | I love hot/cooked food and don't like cold foods | N/A | X | X | X | N/A |
| Participant 7 | Dressed plate with almonds, various berries, and possibly a home plate | N | Healthy oils | N/A | Image with various legumes in cotton bags, looking like a market display | Dried vegetables so minimal spoilage | N | N/A |
| Participant 7 | Dressed plate with almonds, various berries, and possibly a home plate | N | Soft fruits and berries produced in England | N/A | Image with various legumes in cotton bags, looking like a market display | Much vegetable goes to waste | N | N/A |
| Participant 7 | Dressed plate with almonds, various berries, and possibly a home plate | N | Short transport | N/A | Image with various legumes in cotton bags, looking like a market display | and be stored for a very long time | N | N/A |
| Participant 7 | Dressed plate with almonds, various berries, and possibly a home plate | N | Vegan and veggie food | N/A | Image with various legumes in cotton bags, looking like a market display | Superfood | N | N/A |
| Participant 7 | Dressed plate with almonds, various berries, and possibly a home plate | N | Colourful | N/A | Image with various legumes in cotton bags, looking like a market display | No mass packaging | N | N/A |
| Participant 7 | Dressed plate with almonds, various berries, and possibly a home plate | N | Diverse | N/A | Image with various legumes in cotton bags, looking like a market display | reduced food waste | N | N/A |
| Facilitator 1 | Mixed bowl of various plants, vegetables, grains) and fish looks like a typical social media image | Y | Poke Bowl eaten in Italy | N/A | Image of a steak and kidney pie with green beans | most organs of animals are today not eaten by humans but processed for other sources | Y | N/A |
| Facilitator 1 | Mixed bowl of various plants, vegetables, grains) and fish looks like a typical social media image | Y | Diversity of colour | N/A | Image of a steak and kidney pie with green beans | represents beef from a grass-fed system, sustainable beef | Y | N/A |
| Facilitator 1 | Mixed bowl of various plants, vegetables, grains) and fish looks like a typical social media image | Y | Diversity of nutrients | N/A | Image of a steak and kidney pie with green beans | the entire carcase of the animal isn't valued | Y | N/A |
| Facilitator 1 | X | X | X | N/A | Image of a steak and kidney pie with green beans | organs need to get revalued | Y | N/A |
| Facilitator 2 | Assortment of various wonky vegetables (potatoes, carrots, pumpkin, gourds etc.) | Y | Homegrown | N/A | Scotch eggs made from pork sausages | Created from food surplus | Y | N/A |
| Facilitator 2 | Assortment of various wonky vegetables (potatoes, carrots, pumpkin, gourds etc.) | Y | Knows what went into the plants | N/A | Scotch eggs made from pork sausages | Fed to people in need | Y | N/A |
| Facilitator 2 | Assortment of various wonky vegetables (potatoes, carrots, pumpkin, gourds etc.) | Y | Enjoys the process of cooking from scratch | N/A | Scotch eggs made from pork sausages | Created in community | Y | N/A |
| Facilitator 2 | Assortment of various wonky vegetables (potatoes, carrots, pumpkin, gourds etc.) | Y | Tasty due to short storage times | N/A | Scotch eggs made from pork sausages | A blanket statement of meat being less sustainable challenged | Y | N/A |

| 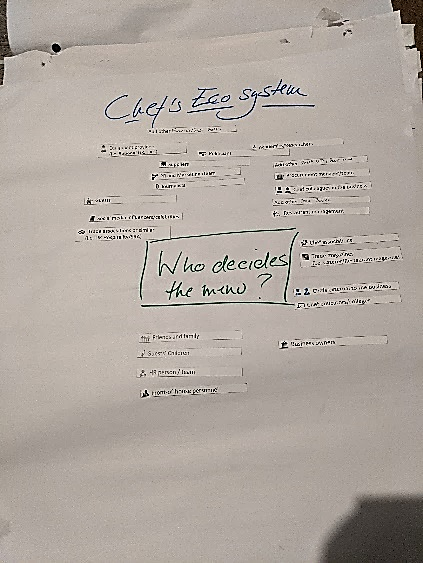 | 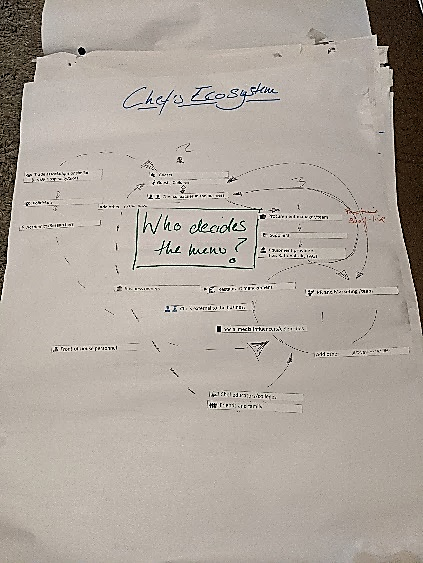 | 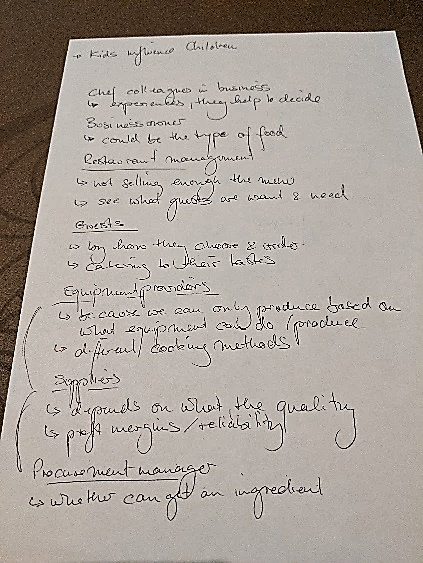 |
| --- | --- | --- |
| Workshop Artefact 8 - Chefs’ Ecosystem Task Group A | Workshop Artefact 9 - Chefs’ ecosystem task Group B | Workshop Artefact 10 - Chefs’ ecosystem task Group B – facilitator notes |
| Alt Text: Flipchart diagram with menu stakeholders arranged in groups. | Alt Text: Flipchart diagram with menu stakeholders arranged in groups with arrows – rudimentary causal loop diagram. | Alt Text: Handwritten notes from the group work of one of the facilitators. |

Table 10 - Stakeholders’ analysis of both groups

| Group A | | | Group B | | |
| --- | --- | --- | --- | --- | --- |
| Stakeholder prompts (prepared by research team) | Stakeholders' participant addition | Distance (1 = very influential - 10 = least influential) | Stakeholder prompts  (prepared by research team) | Stakeholders' participant addition | Distance (1 = very influential - 10 = least influential) |
| Chef colleagues in the business |  | 1 | Chef colleagues in the business | Y | 1 |
| Procurement manager/team |  | 1 | Procurement manager/team | Y | 1 |
| Restaurant management |  | 1 | Restaurant management | Y | 1 |
| Business owners |  | 1 | Politicians | Y | 1 |
| Suppliers |  | 1 |  | (Shelf life / Prep Time*) | 1 |
| Equipment providers (i.e. Rational UK, TAG) |  | 1 | PR and Marketing /team | Y | 2 |
|  | (Cook Books*) | 1 | (Social media influencers/ celebrities) | Y | 2 |
| Chefs external to the business |  | 2 | Suppliers | Y | 2 |
| PR and Marketing /team |  | 2 | Equipment providers (i.e. Rational UK, TAG) | Y | 2 |
| Guests |  | 2 | Academics/ Researchers | Y | 2 |
| Guests’ Children |  | 2 |  | (Finance / Gross Profit*) | 2 |
| Social media influencers/celebrities |  | 3 | Guests | Y | 3 |
| Front-of-house personnel |  | 4 | Journalists | Y | 3 |
|  | (Advertisement*) | 4 | Friends and family | Y | 3 |
| Chef educators/colleges |  | 5 |  | (Cook Books*) | 3 |
| Trade associations or similar (i.e. UK Hospitality/SRA) |  | 5 | Chefs external to the business | Y | 4 |
| Friends and family |  | 5 | Front-of-house personnel | Y | 4 |
| Politicians |  | 5 | Business owners | Y | 4 |
| Academics/Researchers |  | 5 | Guests’ Children | Y | 4 |
| HR person/team | Prompt not used | N/A | Chef associations | Y | 4 |
| Chef associations | Prompt not used | N/A | Chef educators/colleges | Y | 4 |
| Journalists | Prompt not used | N/A | Trade associations or similar (i.e. UK Hospitality/SRA) | Y | 4 |
| Trade magazines (i.e. Caterer/Restaurant magazine) | Prompt not used | N/A | Trade magazines (i.e. Caterer/Restaurant magazine) | Y | 4 |
|  |  |  | HR person/team | Y | 5 |

Table 11 - Consolidated stakeholder analysis

| **Importance of stakeholders agreed in the generative dialogue of the group (descending, top highest influence)** |
| --- |
| Politicians (create the law of the place) |
| Academics (influence policy) |
| Finance/Gross Profit (at least 10%) |
| Suppliers |
| Equipment providers |
| Procurement manager/team |
| Shelf life / Prep time |
| PR and Marketing Team (needs to look good & how to advertise) |
| Journalists |
| Guests |
| Chef Colleagues in the business (sous chefs/ team) |
| Social media influencers/celebrities |
| Restaurant management |
| Cookbooks |
| Trade Associations and Trade Magazines |
| Chefs external to the business |
| Chef Educators / Colleges |
| Friends and family |
| Guests Children |
| Business owners |
| HR Team |
| Front of House Personnel |

| 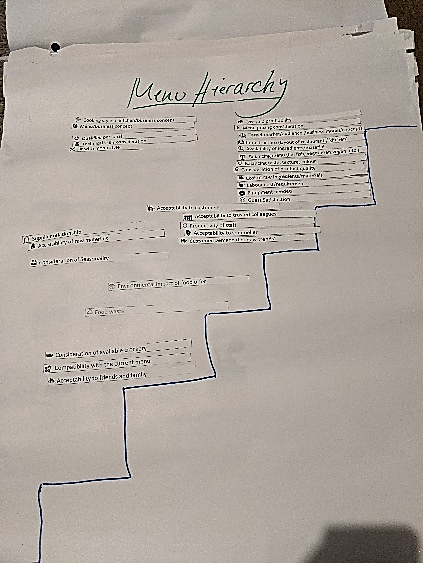 | 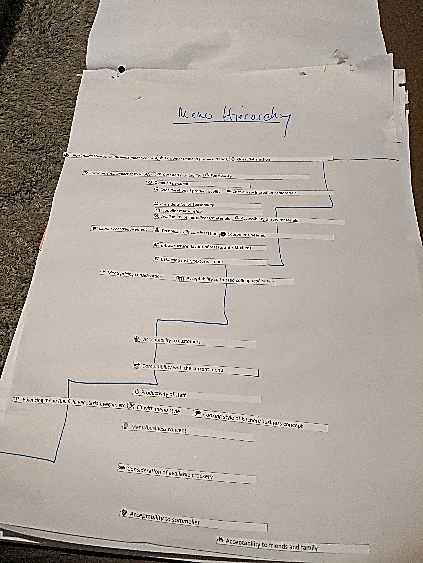 |
| --- | --- |
| Workshop Artefact 11 – Menu hierarchy task Group A | Workshop Artefact 12 - Menu hierarchy task Group B |
| Alt Text: Flipchart displaying a laddered menu hierarchy with grouped influences. | Alt Text: Flipchart displaying a laddered menu hierarchy with relatively dispersed influencing factors. |

Table 12 - Menu hierarchy analysis

| **Group A** | | | **Group B** | | |
| --- | --- | --- | --- | --- | --- |
|  | Selected | Influence (1 = very influential - 5 = less influential) |  | Selected | Influence (1 = very influential - 5 = less influential) |
| Cooking style of kitchen/business concept | Y | 1 | Customer Demand (i.e. new trends) | Y | 1 |
| Cost and profitability | Y | 1 | Guest Satisfaction | Y | 1 |
| Fit with menu style | Y | 1 | Cost and profitability | Y | 1 |
| Infrastructure (layout of restaurant/kitchen) | Y | 1 | Environmental impact of food offer | Y | 1 |
| Menu pricing considerations | Y | 1 | Food waste | Y | 1 |
| Menu/business concept | Y | 1 | Qualified personal | Y | 1 |
| Qualified personal | Y | 1 | Consideration of product quality | Y | 1 |
| Target market/audience (business model/concept) | Y | 1 | Cost of raw ingredients/materials | Y | 1 |
| Technical skills consideration | Y | 1 | Consideration of seasonality | Y | 2 |
| Acceptability to customers | Y | 2 | Supplier relationship | Y | 2 |
| Availability of ingredients/materials | Y | 2 | Accessibility of raw materials | Y | 2 |
| Balancing menu (i.e. fish, vegetarian, vegan, etc.) | Y | 2 | Availability of ingredients/materials | Y | 2 |
| Balancing taste, texture, colour | Y | 2 | Equipment needed | Y | 2 |
| Consideration of product quality | Y | 2 | Labour cost/requirement | Y | 2 |
| Cost of raw ingredients/materials | Y | 2 | Technical skills consideration | Y | 2 |
| Equipment needed | Y | 2 | Infrastructure (layout of restaurant/kitchen) | Y | 3 |
| Guest Satisfaction | Y | 2 | Balancing taste, texture, colour | Y | 3 |
| Labour cost/requirement | Y | 2 | Acceptability to trusted colleagues | Y | 3 |
| Acceptability to sommelier | Y | 3 | Menu pricing considerations | Y | 3 |
| Acceptability to trusted colleagues | Y | 3 | Target market/audience (business model/concept) | Y | 3 |
| Accessibility of raw materials | Y | 3 | Acceptability to customers | Y | 4 |
| Consideration of seasonality | Y | 3 | Compatibility with the current menu | Y | 4 |
| Customer Demand (i.e. new trends) | Y | 3 | Productivity of staff | Y | 4 |
| Productivity of staff | Y | 3 | Balancing menu (i.e. fish, vegetarian, vegan, etc.) | Y | 4 |
| Supplier relationship | Y | 3 | Cooking style of kitchen/business concept | Y | 4 |
| Environmental impact of food offer | Y | 4 | Fit with menu style | Y | 4 |
| Food waste | Y | 4 | Menu/business concept | Y | 5 |
| Acceptability to friends and family | Y | 5 | Consideration of available crockery | Y | 5 |
| Compatibility with the current menu | Y | 5 | Acceptability to sommelier | Y | 5 |
| Consideration of available crockery | Y | 5 | Acceptability to friends and family | Y | 5 |

| 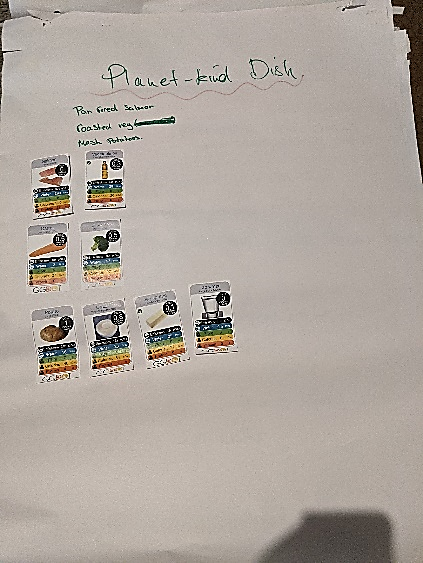 | 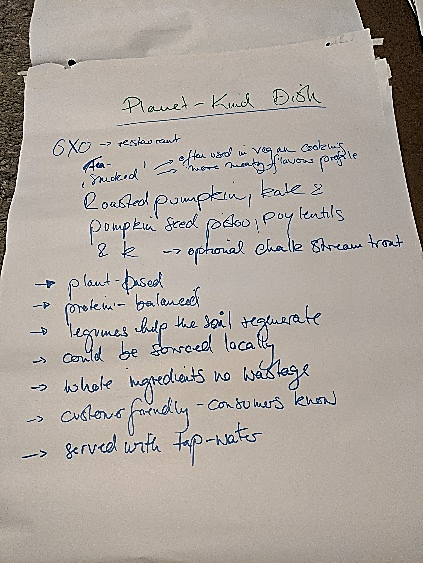 |
| --- | --- |
| Workshop Artefact 13 - Planet kind dish task Group A | Workshop Artefact 14 – Planet kind dish task Group B |
| Alt Text: Flipchart with eight grouped GGDOT flashcards, which are the key ingredients of the proposed dish. | Alt Text: Flipchart with a handwritten dish description and the framing of the proposal. |

Table 13 - Analysis of sustainable dishes

|  | **Group A** | **Group B** |
| --- | --- | --- |
| Name of dish | Pan-fried salmon, roasted vegetables, mashed potatoes | Tea-smoked roasted pumpkin, kale, pumpkin seed pistou, and Puy lentils with optional chalk stream trout |
| Ingredient 1 | Salmon | Tea |
| Ingredient 2 | Vegetable Oil | Pumpkin |
| Ingredient 3 | Carrots | Kale |
| Ingredient 4 | Broccoli | Pumpkin Seeds |
| Ingredient 5 | Potatoes | Oil |
| Ingredient 6 | Cream | Garlic |
| Ingredient 7 | Vegetable spread | Olive oil |
| Ingredient 8 | Water | Fresh basil |
| Ingredient 9 | N/A | Breadcrumbs |
| Ingredient 10 | N/A | Potatoes |
| Ingredient 11 | N/A | Puy lentils |
| Ingredient 12 | N/A | Chalk stream trout |
| Sustainable narrative 1 | Wanted to use the insights gained linked to GHGE | Smoking is often used in vegan cooking to create a meaty flavour |
| Sustainable narrative 2 | Cards were easy to use and allowed us to pick ingredients lower in GHGE | Balanced in protein content |
| Sustainable narrative 3 | Dish for a local restaurant serving the community rather than fine dining | Legumes good for the soil |
| Sustainable narrative 4 | N/A | All can be sourced locally |
| Sustainable narrative 5 | N/A | Use of whole ingredients, no wastage |
| Sustainable narrative 6 | N/A | Customer-friendly - items most guests are familiar with |
| Sustainable narrative 7 | N/A | Served with tap water to avoid further environmental impact |
| Sustainable narrative 8 | N/A | Designed for a fine-dining restaurant |
| Facilitator comment | Observing the group, it appeared they were looking for a route of least resistance, and using the cards felt like a safe choice, as it did not require discussing the more profound complexities and allowed them to have a slightly longer break. | Chefs felt excited about the opportunity to design a dish in this context. |
| Facilitator comment | This was after lunch, and it appeared everyone was pretty tired by then. It would have been beneficial to incorporate some form of energiser. | Touched on the concern that not all guests would consider it an option |
| Facilitator comment | This was the group with an overall more junior group of chefs | Conversation and dialogue were led by more senior chefs in this group, with less input from the junior chefs |
| Facilitator comment | N/A | However, chefs were trying to engage the more junior team members |
| Facilitator comment | N/A | This tension between positions in the kitchen was potentially enhanced because there was a pastry chef in the group who does not usually design savoury dishes. |

| 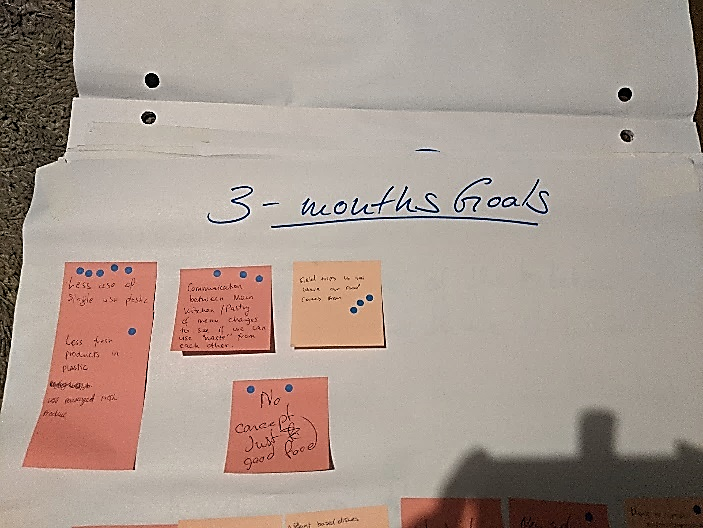 | 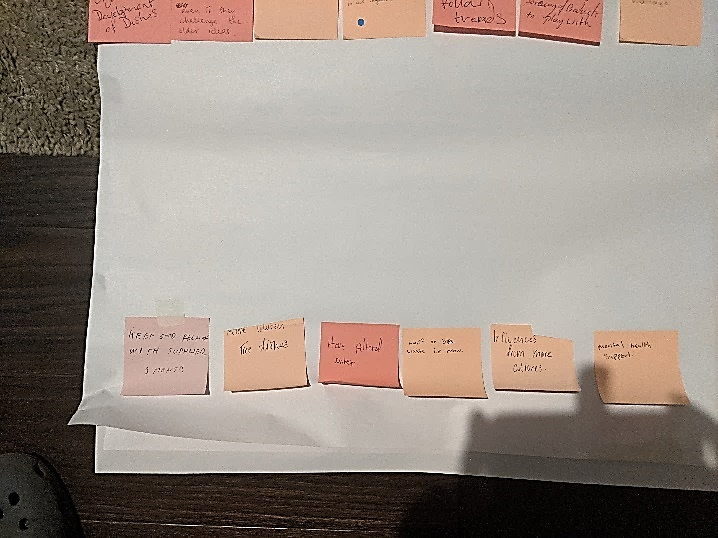 |
| --- | --- |
| Workshop Artefact 15 – Blue-Sky thinking task 3 months top choice | Workshop Artefact 16 – Blue-Sky thinking task 3 months other choices |
| Alt Text: Flipchart top half with six post-its containing proposals for action in 3 months, blue dots used as votes are patterned on the post-its. | Alt Text: Flipchart bottom with six additional post-its for the 3-month actions, but they do not have blue dots on them. |
| 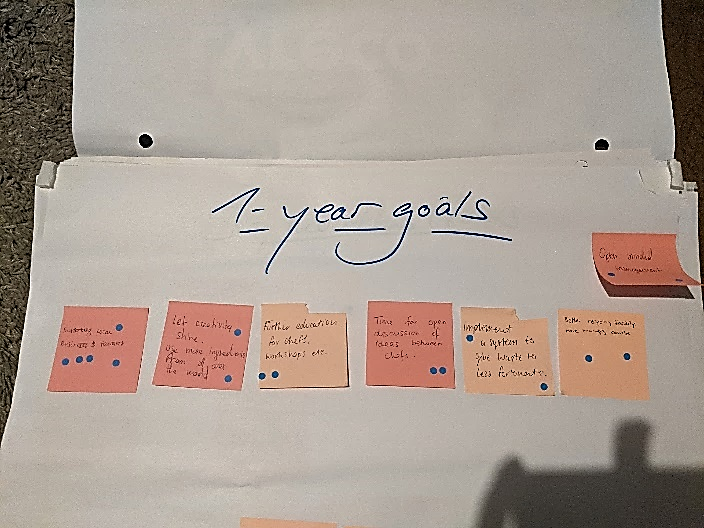 | 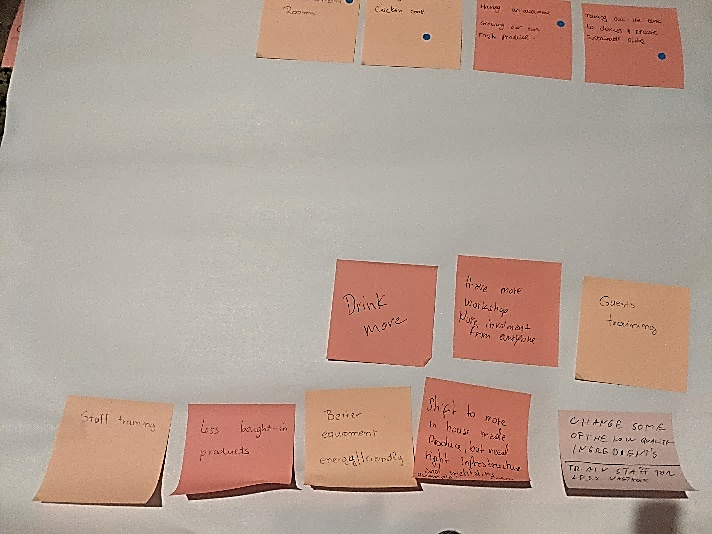 |
| Workshop Artefact 17 – Blue-Sky thinking task 1-year top choices | Workshop Artefact 18 – Blue-Sky thinking task 1-year other choices |
| Alt Text: Flipchart top half with heading ‘1-year-goals’ and seven post-its which have proposed actions on them, each of them has blue-dot stickers from the group's vote on them. | Alt Text: Flipchart bottom half containing eight post-its with proposed actions for 1 year, which have not received any blue dot votes. |
| 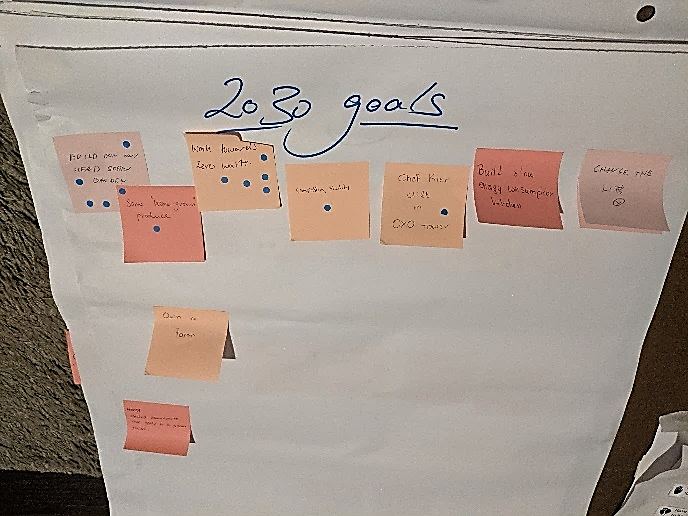 |  |
| Workshop Artefact 19 – Blue-Sky Thinking Task 2030 choices |  |
| Alt Text: Flipchart image with nine post-its and a heading of ‘2030-goals’, five post-its have blue dot votes, the other four do not. |  |

Table 14 - Blue-Sky Thinking Action Priority Analysis

| **3 months' ideas (descending priority)** | **1-year ideas (descending priority)** | **2030 ideas (descending priority)** |
| --- | --- | --- |
| Less use of single-use plastics (buy fewer products in plastic punnets) | Supporting local businesses and farmers | Grow your own produce - herbs and small produce |
| Communication between the main kitchen and pastry for menu changes so we can use by-products from each other’s dishes | Further education for chefs, workshops, etc | Work towards zero waste |
| Field trips to learn where the food comes from | Time for open discussion of ideas between chefs | Compost facility |
| Make it not a concept, but just prepare good food | Let creativity shine using more ingredients from all over the world | Current head chef still in position |
| Not just trends | Implement a system to give surplus to less fortunate | Build a low energy consumption kitchen |
| Organic development dishes | Better recycling facilities, more training courses | Change the lifts |
| New ideas are encouraged even if they challenge the older ideas | Open Minded management | Own a farm |
| Sustainable development team | Fermentation room | reached somewhere in our goals for a greener future |
| Search for new suppliers new products to be able to play with | Herb garden, chicken coop |  |
| More on-hand experimentation with by-products | Having an allotment to grow our own produce |  |
| More plant-based dishes | Taking time out to discuss and create sustainable dishes |  |
| Utilising all aspects of food | Drink more |  |
| Find more creative ways to use ingredients | Have more workshops and more involvement from everyone |  |
| Keep good relations with suppliers. | Guest training |  |
| More gluten-free dishes | Less bought-in products |  |
| Have filtered water | Better equipment -energy-friendly |  |
| Use by-products in pasta and ice creams. | Shift to more in-house made produce, but need the proper infrastructure and mentality - i.e. pasta. |  |
| Influences from more cultures | Change some of the low-quality ingredients. |  |
| Mental health support | Train staff for less wastage |  |

| 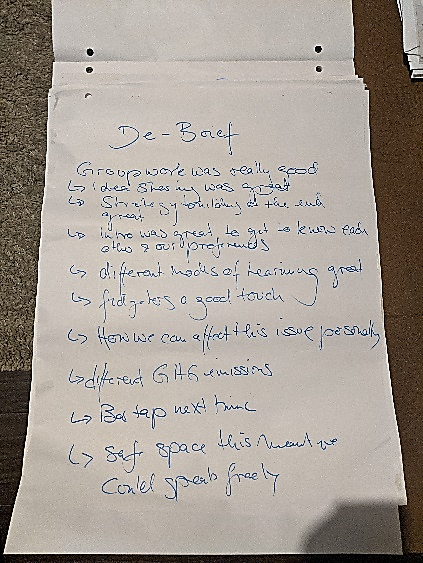 | 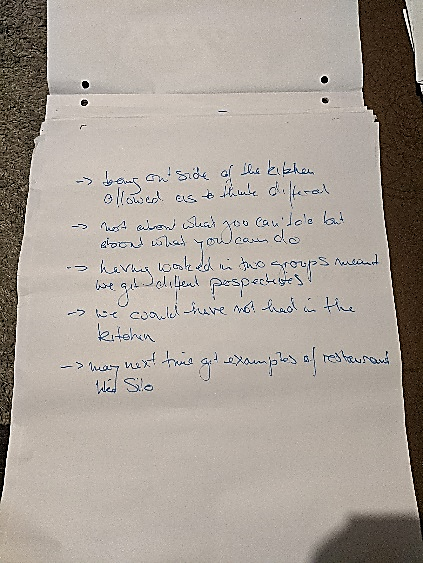 |
| --- | --- |
| Workshop Artefact 20 – workshop debrief flipchart record 1 | Workshop Artefact 21 - – workshop debrief flipchart record 2 |
| Alt Text: Handwritten facilitators' notes on a flipchart titled de-brief. | Alt Text: A second continuation flipchart page of handwritten facilitator notes from the de-brief. |

| 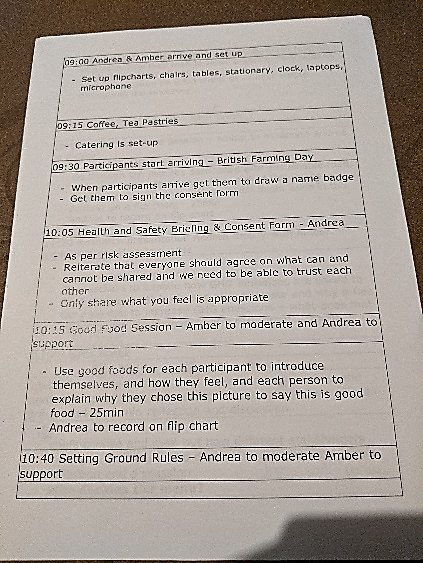 | 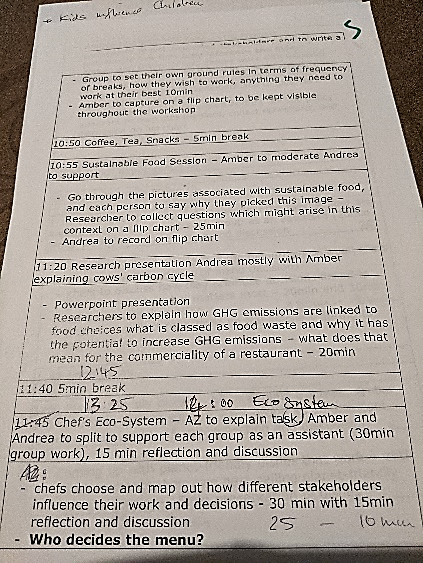 |
| --- | --- |
| Workshop Artefact 22 – facilitator timeline 9 am to 10:40 am | Workshop Artefact 23 – facilitator timeline 10:40 am to 2 pm |
| Alt Text: An image of the printed timeline from 9 am to 10:40 am with no added notes. | Alt Text: An image of the printed timeline from 10:40 to 11:45 am, but with handwritten notes which indicate the timeline was extended to 2 pm. |
| 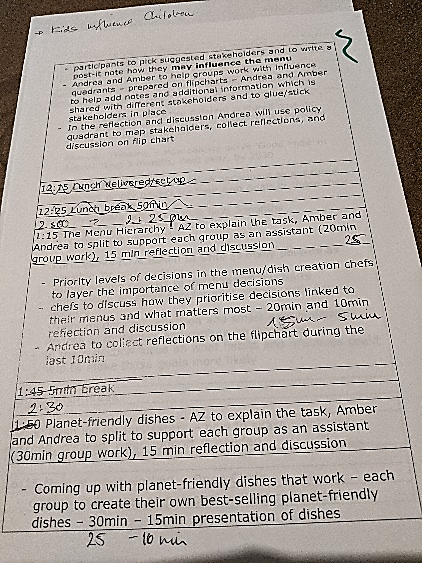 | 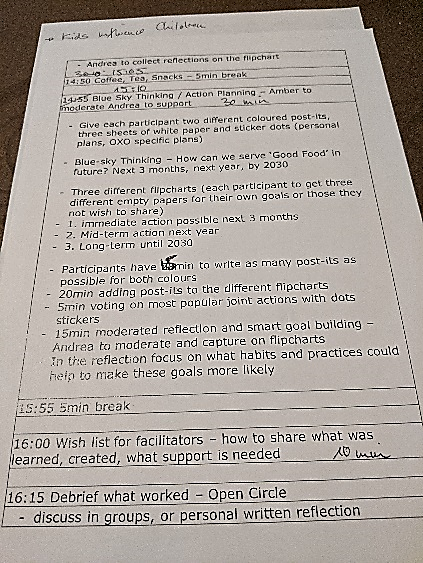 |
| Workshop Artefact 24 – facilitator timeline 2 pm – 2:30 pm | Workshop Artefact 25 – facilitator timeline 2:30 pm to 4:30 pm |
| Alt Text: An image of the printed timeline from 12:15 to 13:50 with handwritten notes indicating the time changes of the day, extending it to 2:30 pm. | Alt Text: An image of the printed timeline from 14:50 to 16:15 with handwritten notes indicating the time changes during the workshop delivery. |

## Appendix 7 Workshop feedback

### Feedback from the in-person debrief

- Group work was really good
- Idea sharing was great
- Strategy building at the end was great
- The introduction, accompanied by good food and sustainable options, worked well to help us get to know each other and our preferences.
- Enjoyed the different modes of learning
- Fidget tools are a nice touch
- It showed us how we can affect this issue personally
- We learned about different GHGE impacts
- I would love to have a bar tab next time
- It was a safe space, which meant we could speak freely
- Being outside the kitchen allowed us to think differently
- This was not about what you can't do but focused on what you can do
- Working in two groups gave us different perspectives, which was useful
- We could not have these perspectives in the kitchen
- Maybe next time, bring along case studies and examples such as Silo

Table 15 - Summary of participant feedback two weeks after the workshop

| **Participant ID** | 001 | 002 | 003 |
| --- | --- | --- | --- |
| **Feedback format** | In-person interview | Written response | In-person interview |
| **Seniority of participants** | Senior chef | CDP | Junior |
| **Thoughts on workshop in general** | It gave the opportunity to speak about why things in the business aren’t always done in a certain way; it helps all of us to think differently. Was very good. The workshop got team members to work together and allowed me (a senior team member) to understand how junior team members think. | The workshop was well-organised, informative, interesting, and a good way to see different points of view from chefs of all levels on the ideas of sustainability and food waste. The mix of participation from everyone, as well as a more structured presentation of information on group tasks, was also well-judged. | It was very insightful. There was a lot to take away for us to do more research on away from work. Everyone brought along their own ideas and how they can make a difference. |
| **Sentiment** | Very positive | Very positive | Very positive |
| **Scene setting and ground rules** | The ground rules and explaining how to work together were useful (albeit me personally not liking rules that much). The facilitators kept the workshop and way to interact at the right level and were approachable throughout. | The setup of the workshop, with an introductory exercise and a welcoming atmosphere, was just right. The idea of using the slips of paper to set up the ground rules for the workshop and how we could all agree/disagree was also good and made way for a safe space to share ideas. | Acceptable ground rules set, easy to follow and understand. |
| **Sentiment** | Positive | Positive | Ok |
| **Pre-workshop task** | Yes, I did enjoy it. It was very important because the stories which were told by the individuals painted a picture of their experiences, and it started the interaction between everyone. | I did enjoy the pre-workshop task as it gave an idea on what topics would be discussed, plus a chance beforehand to gather some thoughts on my current ideas of the terms ‘good food’ and ‘sustainable food’ and provoke some interest in what I felt I was missing in my understanding of those. It was brought into the workshop in an approachable, comfortable way. Everyone had a chance to express freely what they felt on each of their photographs and to talk around their ideas. This meant I could see where each person was coming from and gave me some fresh ideas on how broad each of those terms are as a topic which was interesting. | Yes, because it gave me the chance to do some own research and think about the topics: What is good food and what is sustainable food? |
| **Sentiment** | Very positive | Very positive | Very positive |
| **Researcher presentation** | Yes, it was valuable because it helped us to realise the complexities and also how the scientific narratives change, i.e. different ways of cattle farming. | The information provided was definitely valuable. In particular, the different types of emissions as the main focus is usually ‘just carbon’ where as this showed there are lots of factors to think about for example the cyclic nature of cow farming, plus how land can be reused for multiple purposes. This meant when it came to the group tasks, I felt a bit more informed to make choices, especially for the planet-friendly dish task. | Yes, it did make us think about how far food travels, how much GHGE are embedded and what alternatives there might be. |
| **Sentiment** | Positive | Very positive | Positive |
| **Chefs ecosystem** | It was easy enough, and this exercise was, in my opinion, of particular value to the younger team members as they may have not taken an active role yet in menu development. It also reminded me that not thinking about off-cuts in menu design can lead to more waste and/or more labour costs, and the management doesn’t always listen to this. | The chef’s ecosystem task was interesting as there were differences of opinion in priorities within the group dependent on experience level and background. This meant it wasn’t necessarily easy, but I didn’t consider that a bad thing. It opened up further discussion and meant we could all see that actually there are even more important factors in terms of who decides a menu than we would first think or sometimes hope. It was useful for me at my level to get more of a grasp of the many people and positions involved and influencing a menu choice, particularly in a larger company. I think the idea again of using the small bits of paper with prompts.  And allowing us to add our own if necessary was well done and didn’t need to be done differently. | It wasn’t easy/or difficult; it made us think about who actually comes first when we decide on our menu. Is it guests or the chef… |
| **Ease** | Easy | Ok | Ok |
| **Sentiment** | Positive | Positive | Positive |
| **Improvement suggestion** | None | None | None |
| **Menu priorities** | It was easy enough. At this point, everyone started thinking differently. | Again, this task was useful to me personally and seemed good for the group overall. It was good that this was the second task, following the chef’s ecosystem, as it felt like we had prepared some thoughts of priorities, and the two linked together well. Nothing to do differently. The idea of placing the  Papers down and then rearranging as we added and then finalising at the end worked well. | It wasn’t easy/or difficult, it gave us to think about what we should consider first when writing menus (i.e. easily accessible ingredients, GHGE, where the food comes from, etc.) |
| **Ease** | Easy | Easy | Ok |
| **Sentiment** | Positive | Positive | Positive |
| **Improvement suggestion** | None | None | None |
| **Sustainable dish task** | It was good to be done. It was fun even though we may not always be able to get the specific ingredients we might want to use (i.e. the Carlin peas we were gifted). However, it got people to be a bit more playful and one of the dishes ideated was trialled the next day in the kitchen. | This task made me question each ingredient to be used on a dish and how to get the most out of it, which will be useful to consider in the future. Chefs can have a lot of influence at this point in menu development in creating more planet-friendly dishes. If each chef already has all of the information in mind at the very beginning of coming up with a dish or knows how to substitute for more sustainable ingredients or methods, it can make a significant change in a business. Again, I think this was a well-managed task with some.  Freedom to explore an idea and no pressure for anything concrete or fully formed. Just a  Brainstorming and refining exercises as a group. | Medium difficulty. It gave us a chance to open our minds and hear other people's ideas (i.e. using tea for smoking instead of wood, looking at balancing airmiles against GHGE within a dish). The additional resources and references were very useful, especially the GGDOT Cards (Armstrong *et al.*, 2020). |
| **Ease** | Easy | Easy | Ok |
| **Sentiment** | Positive | Positive | Positive |
| **Improvement suggestion** | I would have gotten the chefs to create the dish in real life. | None | None |
| **Blue-Sky thinking** | For me, this wasn’t particularly useful because sometimes you cannot know what’s going to happen from one week to another because the management and PR might change direction or we might not be able to get certain ingredients, so this only works if the other areas of the business also support this change. | This was a good task to end on and brought the workshop together well. This was easy to do with the  Freedom to move around and place ideas on each Wishlist sheet. It was useful to see that many ideas.  And wish lists were similar and shows that even just a small group could start to make a change as  There are some common goals.   No changes to this part. It was a good way to do it with  Minimum pressure on participants and the voting system was a good idea, too. | Medium difficulty was a good exercise to think outside of the box on how to change the future. There were a lot of common ideas in the group, that was reassuring. Such as how can we make sure we use the full produce. |
| **Ease** | Ok | Easy | Ok |
| **Sentiment** | Not useful | Positive | Positive |
| **Improvement suggestion** | Getting the wider team involved (beyond chefs) | None | None |
| **Was everyone able to freely contribute** | Yes, but it depended on the group. That’s why the group interaction was useful. | It felt like a safe space to share opinions and ideas. The workshop was well run and moved along if  Side-tracked or brought back to someone’s original point if it went off at a tangent, which was  important to make everyone feel valued and included. | Yes |
| **Sentiment** | Positive | Positive | Positive |
| **Was it well paced** | Yes | The pace was good, with breaks being frequent enough for me personally and not too long as to ruin the pace of it. | Yes |
| **Sentiment** | Positive | Positive | Positive |
| **Length of workshop** | No perfect | For me it was the perfect length as it meant a lot was covered but not too quickly and me to think about it as it went along. | Would have loved to stay longer/learn more. |
| **Sentiment** | Very positive | Very positive | Positive |
| **Suitability of venue** | Fabulous, bright and airy. | The venue was nice and not too much of a trek from our usual place of work. It was a shame about the continued works in there causing some noise disruption, but it would work for future workshops. | The location was easy to find, and room was well set up. |
| **Sentiment** | Very positive | Positive | Very positive |
| **Workshop content suitability** | yes | The content was well-mixed, informative and useful for me as a chef. | Yes. It opened everybody's mind to the impact we might have. i.e. should we get strawberries from far away or local? What can we exchange for avocadoes so things we would not speak about at work? |
| **Sentiment** | Positive | Positive | Positive |
| **Anything to add** | No | I think this research is important and valuable. It’s also important to make chefs aware that it is happening and to get involved as we, as a group, do have a way to make a change in the hospitality industry. I would hope that eventually, it becomes a basic level of training within the industry in colleges and workplaces to raise awareness of food waste, GHGE and sustainable practices linked to restaurants and our place in the food industry. Workshops linked to this research will be useful to chefs of all levels. | No |

**References:**

Adhianata, H., Adi, A.C. and Qomaruddin, M.B. (2023), “A preliminary study of chefs’ knowledge and attitude towards nutrition during restaurant’s food handling”, *Journal of Public Health in Africa*, Page Press Publications, Vol. 14 No. S2, doi: 10.4081/jphia.2023.2616.

Ariza-Montes, A., Arjona-Fuentes, J.M., Han, H. and Law, R. (2018), “The price of success: A study on chefs’ subjective well-being, job satisfaction, and human values”, *International Journal of Hospitality Management*, Elsevier Ltd, Vol. 69, pp. 84–93, doi: 10.1016/j.ijhm.2017.10.006.

Armstrong, B., Bridge, G., Oakden, L., Reynolds, C., Wang, C., Panzone, L.A., Rivera, X.S., *et al.* (2020), “Piloting Citizen Science Methods to Measure Perceptions of Carbon Footprint and Energy Content of Food”, *Frontiers in Sustainable Food Systems*, Frontiers Media S.A., Vol. 4, doi: 10.3389/fsufs.2020.00120.

Azar, A.S., Albattat, A. and Kamaruddin, A.Y. (2020), “The effectiveness of hospitality experiential learning from academic and industry perspectives”, *Journal of Critical Reviews*, SynthesisHub Advance Scientific Research, Vol. 7 No. 12, pp. 296–301, doi: 10.31838/jcr.07.12.56.

Balkaran, R. and Giampiccoli, A. (2013), *National Scarce Skills within the Professional Chef’s Sector in South Africa*, Vol. 4.

Batat, W. (2020), “Pillars of sustainable food experiences in the luxury gastronomy sector: A qualitative exploration of Michelin-starred chefs’ motivations”, *Journal of Retailing and Consumer Services*, Elsevier Ltd, Vol. 57, doi: 10.1016/j.jretconser.2020.102255.

Batat, W. (2021), “How Michelin-starred chefs are being transformed into social bricoleurs? An online qualitative study of luxury foodservice during the pandemic crisis”, *Journal of Service Management*, Emerald Group Holdings Ltd., Vol. 32 No. 1, pp. 87–99, doi: 10.1108/JOSM-05-2020-0142.

Belardi, S., Knox, A. and Wright, C.F. (2021), “Too hot to handle? An analysis of chefs’ job quality in Australian restaurants”, *Journal of Industrial Relations* , SAGE Publications Ltd, Vol. 63 No. 1, pp. 3–26, doi: 10.1177/0022185620940375.

Bertoldo, J., Hsu, R., Reid, T., Righter, A. and Wolfson, J.A. (2022), “Attitudes and beliefs about how chefs can promote nutrition and sustainable food systems among students at a US culinary school”, *Public Health Nutrition*, Cambridge University Press, Vol. 25 No. 2, pp. 498–510, doi: 10.1017/S1368980021003578.

Birney, A., Phillips, R. and Taylor, R.M. (2025), *Action Inquiry: A Learning Approach for Systems Change*.

Cameron, D. (2001), “Chefs and occupational culture in a hotel chain: A grid-group analysis”, *Tourism and Hospitality Research*, Vol. 3 No. 2, pp. 103–114, doi: https://doi.org/10.1177/14673584010030020.

Caraher, M., Lang, T. and Dixon, P. (2000), “The Influence of TV and Celebrity Chefs on Public Attitudes and Behavior Among the English Public”, *Journal for the Study of Food and Society*, Informa UK Limited, Vol. 4 No. 1, pp. 27–46, doi: 10.2752/152897900786690805.

Carter, P.L. and Nicolaides, A. (2023), “Transformative learning: An emotional (r)evolution”, *New Directions for Adult and Continuing Education*, Wiley, Vol. 2023 No. 177, pp. 25–36, doi: 10.1002/ace.20476.

Cerasa, A., Sarica, A., Martino, I., Fabbricatore, C., Tomaiuolo, F., Rocca, F., Caracciolo, M., *et al.* (2017), “Increased cerebellar gray matter volume in head chefs”, *PLoS One*, Vol. 12 No. 2, doi: 10.1371/journal.pone.0171457.

Chen, H.T. and Wang, C.H. (2019), “Incivility, satisfaction and turnover intention of tourist hotel chefs: Moderating effects of emotional intelligence”, *International Journal of Contemporary Hospitality Management*, Emerald Group Holdings Ltd., Vol. 31 No. 5, pp. 2034–2053, doi: 10.1108/IJCHM-02-2018-0164.

Cifelli, B., Kurp, J., Clarke, T.B. and Clarke, I. (2020), “A comparative exploration of celebrity chef influence on millennials”, *Journal of Foodservice Business Research*, Routledge, Vol. 23 No. 5, pp. 442–470, doi: 10.1080/15378020.2020.1780188.

Condrasky, M., Ledikwe, J.H., Flood, J.E. and Rolls, B.J. (2007), “Chefs’ Opinions of Restaurant Portion Sizes”, *Obesity*, Vol. 15 No. 8, doi: https://doi.org/10.1038/oby.2007.248.

Cooper, J., Giousmpasoglou, C. and Marinakou, E. (2017), “Occupational identity and culture: the case of Michelin-starred chefs”, *International Journal of Contemporary Hospitality Management*, Emerald Group Publishing Ltd., Vol. 29 No. 5, pp. 1362–1379, doi: 10.1108/IJCHM-02-2016-0071.

Cornwall, A. and Jewkes, R. (1995), “What is participatory research?”, *Social Science and Medicine*, Vol. 41 No. 12, pp. 1667–1676, doi: 10.1016/j.socscimed.2009.11.005.

Diaconeasa, M.C., Popescu, G., Maehle, N., Nelgen, S. and Capitello, R. (2022), “Media Discourse on Sustainable Consumption in Europe”, *Environmental Communication*, Routledge, Vol. 16 No. 3, pp. 352–370, doi: 10.1080/17524032.2021.1999295.

van Dijk, J. (2024), “Craft and Design Practice from an Embodied Perspective”, *Craft and Design Practice from an Embodied Perspective*, Routledge, pp. 183–197.

Van den Eeckhout, P. (2017), “The Parisian Cooks’ Union and Chefs de Cuisine (1880s-1930s). Arch-enemies or Allies?”, *Food and History*, Brepols Publishers NV, Vol. 15 No. 1–2, pp. 229–254, doi: 10.1484/j.food.5.116340.

Escalante, R., Bernardo, M. and Arbussa, A. (2022), “Knowledge Transfer in Haute Cuisine: The Relationship between Chefs as an Enabler Factor”, *Journal of Culinary Science and Technology*, Taylor and Francis Ltd., doi: 10.1080/15428052.2022.2087578.

Filimonau, V., Chiang, C.C., Wang, L.E., Muhialdin, B.J. and Ermolaev, V.A. (2023), “Resourcefulness of chefs and food waste prevention in fine dining restaurants”, *International Journal of Hospitality Management*, Elsevier Ltd, Vol. 108, doi: 10.1016/j.ijhm.2022.103368.

Fischer, K.W. (2009), “Mind, brain, and education: Building a scientific groundwork for learning and teaching”, *Mind, Brain, and Education*, Vol. 3 No. 1, pp. 3–16, doi: 10.1111/j.1751-228X.2008.01048.x.

Fooladi, E., Hopia, A., Lasa, D. and Arboleya, J.C. (2019), “Chefs and researchers: Culinary practitioners’ views on interaction between gastronomy and sciences”, *International Journal of Gastronomy and Food Science*, AZTI-Tecnalia, Vol. 15, pp. 6–14, doi: 10.1016/j.ijgfs.2018.11.003.

Frasso, R., Keddem, S. and Golinkoff, J.M. (2018), “Qualitative methods: tools for understanding and engaging communities”, *Handbook of Community Movements and Local Organizations in the 21st Century*, Springer, pp. 527–549.

Giousmpasoglou, C., Brown, L. and Cooper, J. (2020), “The role of the celebrity chef”, *International Journal of Hospitality Management*, Elsevier Ltd, Vol. 85, doi: 10.1016/j.ijhm.2019.102358.

Graf, K., Cohen, A., Miller, B.S. and Vaghi, F. (2019), “Re-examining the Contested Good: Proceedings from a Postgraduate Workshop on Good Food”, *Gastronomica*, University of California Press, Vol. 19 No. 1, pp. 91–93, doi: 10.1525/gfc.2019.19.1.91.

Graham, D., Ali, A. and Tajeddini, K. (2020), “Open kitchens: Customers’ influence on chefs’ working practices”, *Journal of Hospitality and Tourism Management*, Vol. 45, pp. 27–36, doi: https://doi.org/10.1016/j.jhtm.2020.07.011.

De Guzman, A.B., Mesana, J.C.B. and Roman, J.A.M. (2022), “Examining Chefs’ Social Responsibility (CSR) during the COVID-19 pandemic”, *Anatolia*, Routledge, Vol. 33 No. 3, pp. 404–414, doi: 10.1080/13032917.2021.1951782.

Haddaji, M., Albors-Garrigós, J. and García-Segovia, P. (2017), “Women chefs’ experience: Kitchen barriers and success factors”, *International Journal of Gastronomy and Food Science*, Vol. 9, pp. 49–54, doi: https://doi.org/10.1016/j.ijgfs.2017.06.004.

Henly-Shepard, S., Gray, S.A. and Cox, L.J. (2015), “The use of participatory modeling to promote social learning and facilitate community disaster planning”, *Environmental Science and Policy*, Elsevier Ltd, Vol. 45, pp. 109–122, doi: 10.1016/j.envsci.2014.10.004.

De Jaegher, H. and Di Paolo, E. (2007), “Participatory sense-making: An enactive approach to social cognition”, *Phenomenology and the Cognitive Sciences*, Vol. 6 No. 4, pp. 485–507, doi: 10.1007/s11097-007-9076-9.

Kang, B., Twigg, N.W. and Hertzman, J. (2010), “An examination of social support and social identity factors and their relationship to certified chefs’ burnout”, *International Journal of Hospitality Management*, Vol. 29 No. 1, pp. 168–176, doi: 10.1016/j.ijhm.2009.08.004.

Kohli, N. and Mehta, M. (2022), “Occupational stress: A case study among chefs and kitchen workers”, *International Journal of Advances in Engineering and Management (IJAEM)*, Vol. 4, p. 970, doi: 10.35629/5252-0403970977.

De La Lama, R.L., De La Puente, S. and Valdés-Velásquez, A. (2020), “Bringing sustainable seafood back to the table: Exploring chefs’ knowledge, attitudes and practices in Peru”, *ORYX*, Cambridge University Press, Vol. 54 No. 4, pp. 520–528, doi: 10.1017/S0030605318000273.

Lewis, T. and Huber, A. (2015), “A Revolution in an Eggcup? Supermarket Wars, Celebrity Chefs and Ethical Consumption”, *Food, Culture & Society*, Vol. 2 No. 18, pp. 289–307, doi: 10.2752/175174415X14190821960798.

Luna-Reyes, L.F. and Andersen, D.L. (2003), “Collecting and analyzing qualitative data for system dynamics: Methods and models”, *System Dynamics Review*, Vol. 19 No. 4, pp. 271–296, doi: 10.1002/sdr.280.

Macdonald, C. (2012a), *UNDERSTANDING PARTICIPATORY ACTION RESEARCH: A QUALITATIVE RESEARCH METHODOLOGY OPTION*, *Canadian Journal of Action Research*, Vol. 13.

Macdonald, C. (2012b), “Understanding participatory action research: A qualitative research methodology option”, *Canadian Journal of Action Research*, Vol. 13 No. 2, pp. 34–50.

Mahfud, T., Pardjono and Lastariwati, B. (2019), “Chef’s competency as a key element in food tourism success: A literature review”, *Geojournal of Tourism and Geosites*, Editura Universitatii din Oradea, Vol. 26 No. 3, pp. 1057–1071, doi: 10.30892/gtg.26329-417.

Mapes, G. and Ross, A.S. (2022), “Making privilege palatable: Normative sustainability in chefs’ Instagram discourse”, *Language in Society*, Cambridge University Press, Vol. 51 No. 2, pp. 259–283, doi: 10.1017/S0047404520000895.

Marinakou, E. and Giousmpasoglou, C. (2022), “Chefs’ competencies: a stakeholder’s perspective”, *Journal of Hospitality and Tourism Insights*, Emerald Group Holdings Ltd., Vol. 5 No. 1, pp. 205–229, doi: 10.1108/JHTI-06-2020-0101.

McBride, A.E. and Flore, R. (2019), “The changing role of the chef: A dialogue”, *International Journal of Gastronomy and Food Science*, AZTI-Tecnalia, Vol. 17, doi: 10.1016/j.ijgfs.2019.100157.

Mercer-Mapstone, L., Dvorakova, L.S., Groenendijk, L.J. and Matthews, K.E. (2017), “Idealism, Conflict, Leadership, and Labels: Reflections on Co-facilitation as Partnership Practice”, *Teaching and Learning Together in Higher Education*, Vol. 1 No. 21, pp. 1–8.

Mrusek, N., Ottenbacher, M.C. and Harrington, R.J. (2022), “The impact of sustainability and leadership on the innovation management of michelin-starred chefs”, *Sustainability (Switzerland)*, MDPI, Vol. 14 No. 1, doi: 10.3390/su14010330.

Mutlu, H., Demi̇rçakmak, İ.L. and Doğan, M. (2022), “Menu Engineering in the Restaurant Business: A Study on Kitchen Chefs”, *Journal of Tourism and Gastronomy Studies*, Journal of Tourism and Gastronomy Studies, Vol. 10 No. 4, pp. 3537–3553, doi: 10.21325/jotags.2022.1154.

Obbagy, J.E., Condrasky, M.D., Roe, L.S., Sharp, J.L. and Rolls, B.J. (2011), “Chefs’ Opinions About Reducing the Calorie Content of Menu Items in Restaurants”, *Obesity*, Vol. 19 No. 2, pp. 332–337, doi: https://doi.org/10.1038/oby.2010.188.

Ochago, R., Dentoni, D. and Trienekens, J. (2024), “Unraveling the connection between coffee farmers’ value chain challenges and experiential knowledge: the role of farm family resources”, *Journal of Agricultural Education and Extension*, Routledge, Vol. 30 No. 2, pp. 181–211, doi: 10.1080/1389224X.2023.2169479.

Ottenbacher, M. and Harrington, R.J. (2007), “The innovation development process of Michelin-starred chefs”, *International Journal of Contemporary Hospitality Management*, Vol. 19 No. 6, pp. 444–460, doi: 10.1108/09596110710775110.

Percy, R. (2005), “The contribution of transformative learning theory to the practice of participatory research and extension: Theoretical reflections”, *Agriculture and Human Values*, Springer Netherlands, Vol. 22 No. 2, pp. 127–136, doi: 10.1007/s10460-004-8273-1.

Pereira, L.M., Calderón-Contreras, R., Norström, A. V., Espinosa, D., Willis, J., Lara, L.G., Khan, Z., *et al.* (2019), “Chefs as change-makers from the kitchen: indigenous knowledge and traditional food as sustainability innovations”, *Global Sustainability*, Vol. 2 No. 16, pp. 1–10, doi: 10.1017/S2059479819000139.

Pidd, K., Roche, A. and Kostadinov, V. (2014), “Trainee chefs’ experiences of alcohol, tobacco and drug use”, *Journal of Hospitality and Tourism Management*, Elsevier Ltd, Vol. 21, pp. 108–115, doi: 10.1016/j.jhtm.2014.10.001.

Polak, R., Sforzo, G.A., Dill, D., Phillips, E.M. and Moore, M. (2015), “Credentialed Chefs as Certified Wellness Coaches: Call for Action”, *Eating Behaviors*, Elsevier Ltd, Vol. 19, pp. 65–67, doi: 10.1016/j.eatbeh.2015.06.016.

Pope, H., de Frece, A., Wells, R., Borrelli, R., Ajates, R., Arnall, A., Blake, L.J., *et al.* (2021), “Developing a Functional Food Systems Literacy for Interdisciplinary Dynamic Learning Networks”, *Frontiers in Sustainable Food Systems*, Frontiers Media S.A., Vol. 5, doi: 10.3389/fsufs.2021.747627.

Pratten, J.D. (2003a), “What makes a great chef?”, *British Food Journal*, Vol. 105 No. 7, pp. 454–459, doi: 10.1108/00070700310497255.

Pratten, J.D. (2003b), “The training and retention of chefs”, *International Journal of Contemporary Hospitality Management*, Vol. 15 No. 4, pp. 237–242, doi: 10.1108/09596110310475702.

Presencing Institute. (2024), *MITx U-Lab 1x: Leading from the Emerging Future*, edited by Presencing Institute, 6th ed., U-Lab.

Remans, R., Zornetzer, H., Mason-D’Croz, D., Kugler, C., Thornton, P., Pedersen, C., Cattaneo, F., *et al.* (2024), “Backcasting supports cross-sectoral collaboration and social-technical innovation bundling: case studies in agri-food systems”, *Frontiers in Sustainable Food Systems*, Frontiers Media SA, Vol. 8, doi: 10.3389/fsufs.2024.1378883.

Reynolds, C., Moore, S., Denton, P., Jones, R., Abdy Collins, C., Droulers, C., Oakden, L., *et al.* (2022), *A Rapid Evidence Assessment of UK Citizen and Industry Understandings of Sustainability*, doi: 10.46756/sci.fsa.ihr753.

Richardson, L. and Fernqvist, F. (2022), “Transforming the Food System through Sustainable Gastronomy - How Chefs Engage with Food Democracy”, *Journal of Hunger and Environmental Nutrition*, Taylor and Francis Ltd., doi: 10.1080/19320248.2022.2059428.

Robinson, R.N.S. and Barron, P.E. (2007), “Developing a framework for understanding the impact of deskilling and standardisation on the turnover and attrition of chefs”, *International Journal of Hospitality Management*, Vol. 26 No. 4, pp. 913–926, doi: 10.1016/j.ijhm.2006.10.002.

Robinson, R.N.S., Solnet, D.J. and Breakey, N. (2014), “A phenomenological approach to hospitality management research: Chefs’ occupational commitment”, *International Journal of Hospitality Management*, Elsevier Ltd, Vol. 43, pp. 65–75, doi: 10.1016/j.ijhm.2014.08.004.

Scharmer, O.C. (2016), *Theory U: Leading from the Future as It Emerges*, Berrett-Koehler Publishers.

Schnitzler, T. (2020), “Success factors of transformative learning: putting theory into practice”, *Reflective Practice*, Routledge, Vol. 21 No. 6, pp. 834–843, doi: 10.1080/14623943.2020.1821635.

School of Systems Change. (2025), “Spark”, *Webpage*, available at: https://schoolofsystemchange.org/courses/spark/autumn-2024 (accessed 14 February 2025).

Swift, D., Malek, K. and Swift, A. (2019), “The Differences Between ACF Professional Chefs and Chef Educators: Concern for Task Versus Concern for People”, *Journal of Hospitality and Tourism Education*, Routledge, Vol. 31 No. 2, pp. 87–98, doi: 10.1080/10963758.2018.1485496.

Walter, M. (1993), “Participatory Action Research”, 37th ed., Rehabilitation Counseling Bulletin, pp. 1–7.

Wan, T.H., Hsu, Y.S., Wong, J.Y. and Liu, S.H. (2017), “Sustainable international tourist hotels: the role of the executive chef”, *International Journal of Contemporary Hospitality Management*, Emerald Group Publishing Ltd., Vol. 29 No. 7, pp. 1873–1891, doi: 10.1108/IJCHM-08-2015-0406.

Wellton, L., Jonsson, I.M. and Svingstedt, A. (2019), “‘Just trained to be a chef, not a leader’: A study of head chef practices”, *International Journal of Hospitality and Tourism Administration*, Routledge, Vol. 20 No. 4, pp. 400–422, doi: 10.1080/15256480.2017.1397584.

Zuber-Skerritt, O. (2015), “Participatory Action Learning and Action Research (PALAR) for Community Engagement: A Theoretical Framework”, *Educational Research for Social Change*, Vol. 4 No. 1, pp. 5–25.
